# What’s in a Moment: What Can Be Learned About Pair Bonding From Studying Moment-To-Moment Behavioral Synchrony Between Partners?

**DOI:** 10.3389/fpsyg.2020.01370

**Published:** 2020-08-04

**Authors:** Nora H. Prior

**Affiliations:** Department of Psychology, University of Maryland, College Park, MD, United States

**Keywords:** synchrony, coordination, calls, parent, couple, nest

## Abstract

Our understanding of the behavioral and physiological mechanisms of monogamy largely comes from studies of behavioral interactions unique to pair-bonded individuals. By focusing on these highly marked behaviors, a remarkable conservation in the mechanisms underlying pair bonding has been revealed; however, we continue to know very little about the range of behavioral and neurobiological mechanisms that could explain the great diversity of pair-bonding phenotypes that exists both within and across species. In order to capture the dynamic nature of bonds over time and across contexts, we need specific, operationally-defined behavioral variables relevant across such a diversity of scenarios. Additionally, we need to be able to situate these behavioral variables within broader frameworks that allow us to interpret and compare patterns seen across species. Here I review what is known about behavioral synchrony with respect to pair bonding and discuss using synchrony as such a variable as well as a framework to expand on our understanding of pair bonding across timescales, contexts and species. First, I discuss the importance of behavioral synchrony and parental coordination for reproductive success in monogamous biparental bird species. Second, I highlight research documenting the critical importance of interpersonal coordination for human social relationships. Finally, I present recent work that experimentally bridges these lines of research by quantifying moment-to-moment behavioral synchrony during brief social interactions in zebra finch dyads. All together, these distinct perspectives support the notion that synchrony (1) is a shared premise for sociality across species, (2) is deeply shaped by social experiences, and (3) exists across timescales, behaviors, and levels of physiology. Conceptualizing pair bonding through the framework of behavioral synchrony is likely to facilitate a deeper understanding of the nuances of how social experiences and interactions impact the brain and behavior.

## Introduction

### Monogamy and Pair Bonding

The word *monogamy* permeates scientific and popular literature on animals, including humans (Wundt, 1894; Wickler and Seibt, 1981; Dewsbury, 1988). Monogamy is found across species. While it is relatively rare in mammals (4–5%) (Kleiman, 1977), it is more common in primates (∼15%) (Díaz-Muñoz and Bales, 2016) and is the dominant breeding strategy in birds (∼90%) (Lack, 1968; Silver, 1983; Silver et al., 1985). Monogamy is also found in insects (Nalepa and Jones, 1991; Jaffé et al., 2014), lizards (Bull, 2000), and fish (Andrew DeWoody et al., 2000; Morley and Balshine, 2002; Whiteman and Côté, 2004). Traditionally the term monogamy has been used to refer to an “absolute commitment” between a male and female: the male and female breed exclusively with each other, both participate in parental care, remain completely committed social partners, and benefit from reduced sexual conflict (Wundt, 1894; Wickler and Seibt, 1981; Dewsbury, 1988). This monogamous partnership between a male and female is referred to as a pair bond. However, monogamy as a reproductive strategy has resulted from various evolutionary trajectories and is correspondingly diverse; thus, our understanding of monogamy is continually being re-evaluated and developed (Gowaty, 1996; Reichard, 2003; Reichard and Boesch, 2003; Lukas and Clutton-Brock, 2013; Díaz-Muñoz and Bales, 2016; Tecot et al., 2016).

Many of the behavioral interactions between monogamous partners are highly marked, pair-specific expressions of affiliation seemingly resulting from, and reserved for, a pair bond. These highly marked, exclusive pair-directed interactions may be obvious during courtship and initial pair bond formation (Wachtmeister, 2001; Soma and Garamszegi, 2015; Manica et al., 2016; Ota et al., 2018) as well as during the coordination of parental duties (Mariette, 2019). Additionally, for territorial or non-gregarious species the monogamous partnership may be the primary affiliative relationship. For example, selective affiliation for a mate and increased aggression toward novel opposite-sex individuals is used to classify the presence/absence of a pair bond in prairie voles (*Microtus ochrogaster*) (Williams et al., 1992; Resendez et al., 2016). The vast majority of research on the behavioral and physiological mechanisms of pair bonding has taken advantage of these highly-marked examples of monogamy, and this approach has successfully revealed remarkable conservation of key behavioral and neurobiological mechanisms of pair bonding across species. These behavioral and neural mechanisms have been foundational for our understanding of pair bonding across taxa (reviewed in Young and Wang, 2004; O’Connell and Hofmann, 2012; Donaldson and Young, 2016).

Our ever-expanding understanding of the complexity of pair bonding is evident from the changing definition of monogamy itself. Traditionally, monogamy was used to describe partnerships in which individuals have a single, exclusive sexual partner, referred to as genetic monogamy (Wundt, 1894; Wickler and Seibt, 1981; Dewsbury, 1988). However, we now know that genetic monogamy is rare: both within and across breeding periods males and females employ flexible mating strategies (Reichard, 2003; Díaz-Muñoz and Bales, 2016). Indeed, the vast majority of monogamous species are serially (sequentially) socially monogamous, having a single partner at a time, but multiple partners over a lifetime (Wickler and Seibt, 1981; Reichard, 2003). For example, humans may be classified as serially monogamous (Mulder, 2009), and most songbird species form new, transient bonds with each subsequent breeding season (Ens et al., 1996). Even within a breeding season, it is common for both males and females to participate in extra-pair courtship. While the pervasiveness of extra-pair mating was discovered in songbirds, it is also seen in mammals, including the prairie vole (Solomon et al., 2004; Ophir et al., 2008) and several species of primates (Díaz-Muñoz and Bales, 2016). Today, the term monogamy is predominately used to describe cases where a male and female cohabit, referred to as social monogamy (Black and Hulme, 1996; Reichard, 2003).

Research on both the behavioral and neurobiological underpinnings of monogamy has contributed to our understanding of the great diversity in pair bond phenotypes. There is growing evidence that the neurobiological mechanisms supporting pair bonds change over time, particularly between formation and maintenance (Aragona et al., 2006; Prior and Soma, 2015; Resendez et al., 2016; Scribner et al., 2019). More broadly, the neurobiology of social bonds also varies by relationship type (Beery et al., 2008, 2009, 2018). Behaviorally, both within and across species, pair bonds vary in many dimensions including duration and apparent strength of the bond (Black and Hulme, 1996; Tecot et al., 2016). Additionally, although biparental care is typically associated with monogamous mating systems, it is neither ubiquitous nor uniform. For species that do display biparental care, there is significant variation in how parental duties are shared between the male and female (Clutton-Brock, 1991; Hughes, 1998; Cockburn, 2006). Given the tremendous diversity in monogamy, it has even been argued that the term social monogamy is too general, capturing too many distinct phenotypes to be useful (Tecot et al., 2016). Considering the remarkable variability in monogamy within and across species, it stands to reason that highly marked behavioral interactions of pair bonds only represent a small subset of bonds and contexts. In other words, while we have a clear sense of the shared biological basis of pair bonding, we know very little about the behavioral and neurobiological mechanisms underlying the full range of social bonds and diversity in pair bonding phenotypes.

### Challenges in Studying Pair Bonding

There are many challenges when it comes to extending our understanding of monogamy to encompass the diversity that exists across pair bonds. One challenge is that many of the highly marked behavioral variables described above are not applicable across species or contexts. An example of this problem across species can be demonstrated by attempting to apply behavioral variables from certain key model systems to other systems. For example, partner preference is a widely used behavioral metric for identifying monogamously bonded pairs; however, partner preference is not a clear indicator of pair bonds in all species (Prior et al., 2013). Rather, selective affiliation may be specific to modality and context (Gill et al., 2015; Fernandez et al., 2017). Even within a species, marked courtship displays are often absent, rare, or dramatically reduced in intensity after initial pair bonding. In other cases, the specific affiliative behaviors that are necessary for the formation of pair bonds may not be necessary for the maintenance of those bonds (Tomaszycki and Adkins-Regan, 2005, 2006). Overall, it has been particularly challenging to identify changes in affiliative behavior following the formation of a pair bond (Williams et al., 1992; Carter, 1998; Resendez et al., 2016; Scribner et al., 2019). Current research on pair-bond maintenance often requires interrogation of relatively subtle behavioral dynamics (Prior et al., 2016, 2019; Scribner et al., 2019).

A second challenge is how to disentangle the role of parental behavior and biparental care from pair bonding. As indicated above, the extent to which parental duties are shared varies both within and across species. Furthermore, for species that form and actively maintain life-long pair bonds, pair-directed behavior during breeding periods and non-breeding periods can be quite different (Black and Hulme, 1996), thus it is unclear how to compare pair bonds between breeding and non-breeding periods. Not only is this another case in which highly marked behavioral interactions may not be relevant across species and contexts, but this confound raises another set of challenges. First, across species, reproductive behaviors occur under specific neuroendocrine states, during which hormones have profound effects on brain and behavior in order to orchestrate breeding behavior and physiology. This observation raises the question of whether there are distinct neurobiological mechanisms underlying pair-directed behavior during breeding and non-breeding periods. Conversely, it is also important to know how the neuroendocrine conditions associated with breeding impact the expression of the behavioral metrics associated with pair bonding (Prior and Soma, 2015). Furthermore, the confound of biparental care raises questions about how we conceptualize a successful, or strongly bonded, partnership. From an evolutionary perspective, monogamy is a breeding strategy, and evaluation of monogamous partnerships requires assessment of an individual’s reproductive success. There is evidence, however, that for monogamous species reproductive success is related to behavioral, not genetic, compatibility between partners (Ihle et al., 2015), and for species that form long-term pair bonds, reproductive success increases with time (Griggio and Hoi, 2011). This highlights the importance of assessing reproductive success across an individuals’ lifespan and raises the question of how a pair’s experience outside of breeding cycles impacts reproductive success.

A third challenge comes from the fact that not all pair-directed affiliative behaviors are equal. This is true both with respect to the functional significance of affiliative behaviors for a pair bond as well as the value of a behavioral metric of pair bonding for researchers. For example, the same affiliative behaviors used to assess the strength or quality of a bond are also often used to identify the presence of a pair bond initially. By this reasoning, it may be assumed that strongly bonded pairs display more affiliative behaviors across domains. However, various disruptions (e.g., brief stressors, pharmacological and hormonal manipulations) often affect one type of affiliative behavior and not another (Prior et al., 2014), or affect different types of behaviors in opposing ways (Prior et al., 2016, 2018). Such experiments raise the question of how different behavioral components contribute to the formation and maintenance of pair bonds as well as how to assess the consequences of such perturbations on pair bonds. Indeed, there is evidence that certain pair-directed behaviors may be more important for monogamous partnerships [e.g., allopreening (Kenny et al., 2017)].

In order to address these three challenges, we need to identify meaningful dependent variables that can be used to assess the dynamic nature of bonds over time and across contexts (both during breeding and non-breeding periods) and that can support comparisons across species. Because pair bonding fundamentally requires individuals to respond to and align with each other’s behavior, I have turned to behavioral coordination or synchrony as a fundamental behavioral “unit” necessary for understanding social bonding. Behavioral synchrony has specific operational definitions that may be applied across types of affiliative behaviors, timescales, and social contexts. More broadly, behavioral synchrony could be applied as a framework with which to interpret changes or differences in subtle aspects of pair-directed affiliation. Importantly, although behavioral synchrony has been studied across taxa, including insects, fish, birds, and mammals (Bernieri and Rosenthal, 1991; Feldman, 2012b; Duranton and Gaunet, 2016), research on behavioral synchrony is faced with its own set of challenges. Understanding the challenges associated with studying behavioral synchrony itself is necessary in order to determine how behavioral synchrony may be used as a variable to deepen our understanding of pair bonding.

## A Note on Methods: Behavioral Synchrony

Behavioral coordination is inextricably linked with sociality, and synchrony is a ubiquitous component of that coordination. Behavioral coordination/synchrony is essential for a wide range of behaviors including: schooling/flocking (Boinski and Garber, 2000; Greenberg, 2001), group living (Conradt and Roper, 2005; Focardi and Pecchioli, 2005), hunting (Handegard et al., 2012; Bailey et al., 2013), and heterospecific communication [reviewed in Duranton and Gaunet (2016)]. In general it is clear that behavioral synchrony promotes social cohesion (Pays et al., 2007; King and Cowlishaw, 2009), affiliation (Sakai et al., 2010), and prosocial behavior (Van Baaren et al., 2004; Ashton–James et al., 2007; Gueguen et al., 2009) (reviewed in Duranton and Gaunet, 2016). One of the most significant challenges exists in operationalizing synchrony or coordination.

Here I use the term *behavioral synchrony* broadly to encompass the temporal and/or spatial coordination of behaviors as well as physiological and biological states during social interactions. In ethology, it has been proposed that behavioral synchrony has multiple components, including local synchrony (being in the same place at the same time), temporal synchrony (switching actions at the same time), and allelomimicry (engaging in the same behavior at the same time) [reviewed in Duranton and Gaunet (2016)]. In human research, the terms *interpersonal coordination* or *motor-sensory interpersonal synchrony* (individuals moving together and receiving the same sensory stimulation at the same time) are used to capture the integrated nature of bio-behavioral coordination (Bernieri and Rosenthal, 1991; Rennung and Göritz, 2016). Importantly behavioral synchrony is not limited to two individuals (dyads). For example, it is critical for understanding group dynamics such as schooling/flocking (Boinski and Garber, 2000; Greenberg, 2001) and colony living (Conradt and Roper, 2005; Focardi and Pecchioli, 2005). However, for the purpose of this review, I focus on research that investigates behavioral synchrony in social dyads. Furthermore, as a fundamental component of social behavior, many lines of research across disciplines are relevant to investigations of behavioral synchrony. This focus is particularly relevant for animal behavior research, where the terms behavioral synchrony and coordination are less pervasive than in human research. For example, spatial proximity or coordinated activities, which are commonly used dependent variables (Prior et al., 2014, 2016, 2018; Prior and Soma, 2015), are not referred to in the literature as measures of synchrony, despite that they would be classified as synchrony by the above definitions. Considered generally, spatial proximity itself is a hallmark of pair bonding and social bonding in birds and other species (Black and Hulme, 1996; Frigerio et al., 2001; Emery Thompson, 2019; Szipl et al., 2019); but such research lines are not included here.

Furthermore, given that behavioral synchrony promotes social cohesion (Pays et al., 2007; King and Cowlishaw, 2009), affiliation (Sakai et al., 2010), and prosocial behavior (Van Baaren et al., 2004; Ashton–James et al., 2007; Gueguen et al., 2009), it is easy to assume that behavioral synchrony must be positively correlated with pair bonding. Certainly the importance of synchrony in terms of temporal alignment and turn-taking is evident in highly marked behavioral interactions associated with monogamy such as vocal duetting (Hall and Magrath, 2007; Hall, 2009; Odom and Omland, 2017), courtship (Ota et al., 2015) and territorial displays (Ręk and Wong, 2017). However, in many cases these behavioral interactions are specific to key contexts, again leaving it unclear whether this type of coordination is important to pair bonds more generally. Additionally, increased coordination during these marked interactions may not afford any advantage to the partnership (Takeda et al., 2018). In fact, there has been very little research interrogating the relationship between behavioral coordination and pair bonding. At this time there is no clear evidence that behavioral synchrony is specialized or enhanced in monogamous partnerships, and it remains unclear whether synchrony is important for the formation and maintenance of pair bonds or whether variation in the pattern or extent of synchrony is of consequence for monogamous partnerships.

I situate this review within two extensive bodies of work illustrating the importance of moment-to-moment synchrony for monogamous partnerships. First, drawing from rich lines of work in behavioral ecology, I introduce evidence that behavioral synchrony “scales up.” More specifically, I describe the phenomenon that the coordination of parental duties (over the course of hours to days) is achieved during subtle behaviors within brief social interactions. This “active negotiation” of parental duties introduces the notion of behavioral synchrony, alignment, and coordination during brief moments as a type of information exchange. Additionally, I work to address the challenges raised above regarding the confounds that result from focusing on monogamous partnerships during breeding periods. Second, I discuss the extensive literature from human research across the fields of psychology, sociology and anthropology, that have worked to operationalize interpersonal coordination during brief social interactions. This research elegantly integrates and extends the concept of synchrony beyond the behavior of individuals to peripheral physiology and neurobiology. Additionally, this work introduces the importance of our ability to perceive synchrony. In humans, we have a remarkable intrinsic capacity to assess interpersonal coordination of others and the consequences of interpersonal synchrony. Third, I present some of my recent work aimed at experimentally bridging these perspectives from behavioral ecology and social psychology. This work demonstrates the role of sex, social context and social experience in behavioral synchrony by quantifying moment-to-moment behavioral synchrony during brief social interactions in songbird (zebra finch) dyads (Prior et al., 2019, 2020).

Combined, these three areas of research highlight that behavioral synchrony (1) is a shared premise for sociality across species, (2) is deeply shaped by social experience and (3) can be assessed across timescales and behavioral/physiological levels. Importantly, while there is abundant evidence supporting the notion that behavioral synchrony is a fundamental component of monogamous partnerships, there is little evidence that behavioral synchrony is unique to, specialized for, or enhanced in monogamous partnerships. I discuss the significance of this apparent incongruity in the general discussion at the end of the manuscript.

## Behavioral Coordination and Biparental Care in Birds

For the majority of monogamous bird species, the male and female partner share parental duties. However, both within and between species there is substantial variation in the extent to which parental duties are shared. Across many species, parental duties appear to be actively negotiated at the level of the pair. This active negotiation takes place during brief social interactions at the nest, both during incubation (temperature regulation) and nestling provisioning (feeding of chicks). These social interactions often involve dynamic vocal exchanges that are modulated by nesting phase, individual identity as well as by other factors (e.g., presence of a predator) (Mainwaring and Griffith, 2013; Mariette, 2019). The evidence suggesting that these brief social interactions are important for the coordination of parental duties comes from a range of avian species in which characteristics of these brief social interactions have been related to the coordination of parental duties and/or reproductive success of the pair.

There is substantial research from the fields of behavioral and evolutionary ecology on the function of parental coordination across many species of birds. Here, I summarize several of these lines of work, identifying: (1) how behavioral “coordination” or “synchrony” is operationally defined (during brief social interactions and during parental behavior); (2) the behavioral mechanisms (e.g., characteristics of the brief social interactions) that are thought to be involved in the active negotiation of behavioral coordination; and (3) how behavioral measures of coordination have been related to measures of reproductive success or pair bond success. Given the variation in parental behavior across species and by reproductive stage, I summarize these lines of work separately for incubation and provisioning of chicks. Additionally, I work to highlight the species-specific ecologies that influence patterns of parental coordination, the mechanisms by which parental duties are coordinated, and the consequences of parental coordination.

### Incubation

The primary goal of parental care during incubation is to control the thermal environment of the eggs. In many species, the female predominately incubates the eggs whereas in others the parents divide incubation duties equally. In species where the male does not incubate, he may perform other duties such as provisioning the female and/or acting as a sentinel and alerting the female to nest predators.

Some of the earliest evidence demonstrating that monogamous partners are actively negotiating and coordinating parental care duties came from research on ring doves (*Streptopelia risoria*). Female ring doves do the majority of the incubation, whereas males typically contribute incubation relief during the day. The extent of this incubation relief varies greatly from pair to pair as males have been observed to incubate 23–76% of the time (Wallman et al., 1979). This sharing of parental duties is a pair-level phenotype, not entirely driven by the male or female: the proportion of time each partner incubates changes when they are re-paired with a different mate during a new breeding cycle (Wallman et al., 1979). The majority of these incubation exchanges (over three quarters) are coordinated, meaning they are initiated by the incubating parent and leave no gap in incubation (Ball and Silver, 1983; Silver et al., 1985). The most common behavioral exchange associated with this coordinated transition is a brief allopreening bout (almost half of exchanges) (Ball and Silver, 1983). This coordination between partners does not appear to be caused simply by physiological synchrony around the nesting cycle, because switching partners between nests at the same breeding point (thus in the same physiological condition) within a breeding season causes significant disruptions in the timing of incubation bouts, and changes patterns of parental interactions (Ball and Silver, 1983). This experimental evidence is consistent with the notion that the coordination of parental behavior is an emergent consequence of the behavioral interactions between partners.

Female great tits (*Parus major*) also are directly responsible for much of the parental care: they build the nests, incubate the eggs, and brood hatchlings largely alone (Cramp and Perrins, 1982). Male great tits contribute to parental care by provisioning the female with food while she incubates (Hinde, 1952). Interestingly, the male coordinates his provisioning behavior with the female. During this period, the male and female have brief vocal exchanges where the male sings from a perch and the female answers predominately with calls (Gorissen and Eens, 2004; Boucaud et al., 2016b, c). These vocal exchanges are longer and more rapid when the male feeds the female (Boucaud et al., 2016c), and experimentally manipulating food availability shows that females use calling as an honest indicator of their hunger levels (Boucaud et al., 2016b). These lines of work emphasize that much behaviorally relevant information can be communicated in these coordinated nesting exchanges.

Male and female zebra finches (*Taeniopygia guttata*) share incubation duties relatively equally. Again this sharing of duties is an active process which appears to be negotiated via calling exchanges (Elie et al., 2010; Boucaud et al., 2016a, 2017; Villain et al., 2016). These interactive vocal exchanges, similar to “duets,” were originally described in wild breeding zebra finches (Elie et al., 2010). There are two main types of vocal exchanges, both of which can be initiated by either the male or female. “Meeting sequences” are the dominant vocal exchange during incubating, occurring when one partner returns to the nest, which may or may not result in a nesting exchange (or nesting relief). During “sentinel sequences” one partner is perched outside the nest and the pair has a brief vocal exchange, which seems to be related to shared vigilance and nesting defense (Elie et al., 2010; Mainwaring and Griffith, 2013). Both types of vocal sequences are very brief (1-2 minutes on average) and both types are characterized by tight temporal coordination and alternation of calling between partners (Elie et al., 2010). In wild zebra finch pairs it has been demonstrated that characteristics of these vocal exchanges predict whether or not a nest exchange (or relief) will occur; specifically, the female’s call rate and the acoustic structure of her calls predicts whether or not the male performs a nesting relief (Boucaud et al., 2017). In captive zebra finches, the timing of incubation bouts has been experimentally manipulated via delaying the male partners’ return to the nest, thus extending the female’s incubation bout (Boucaud et al., 2016b). Interestingly, call rate and the acoustic structure of calls during the nesting relief following this disruption are significantly affected by the delay and predicts the duration of the female’s subsequent time off the nest (Boucaud et al., 2016b). Together with the work in great tits, these lines of work emphasize the range of ways birds can coordinate activities using dynamic vocal exchanges at the nest.

Some species also vary in the proportion of pairs that form any type of monogamous partnership. The northern lapwing (*Vanellus vanellus*) is a biparental shorebird that forms monogamous partnerships about 80% of the time (Liker and Székely, 1999). The male’s contribution to incubation is highly variable (Liker and Székely, 1999). Typically, incubation exchanges occur at “exchange gaps” and thus are not coordinated exchanges. Having exchange gaps during incubation is not uncommon among shore and seabirds (Niebuhr and McFarland, 1983; Bulla et al., 2013). Prior to departing from the nest, females, but not males, perform vocal displays which appear to signal the male. Female vocalizing increases the likelihood that the male will incubate and decreases the duration of the exchange gap (Sládeček et al., 2019). Despite significant species differences in how incubation duties are shared, the range of ways birds coordinate activities using dynamic vocal exchanges at the nest remains striking.

### Provisioning Nestlings

Whereas there is significant variation in how incubation duties are shared, there is much greater consistency across species in biparental care around nestling provisioning. The coordination of nestling provisioning can be described in several ways. Parents can either alternate or synchronize the timing of their visits. Synchronization between parents occurs when both parents visit the nest at the same time (typically defined as entering the nest within 1–2 min of each other). This coordination is consistent broadly with the definition of activity synchrony (Duranton and Gaunet, 2016). Parents can also synchronize their foraging trips.

In the vast majority of species studied, parents synchronize a majority of their nest visits and alternate their feeding trips more than would be expected by chance [great tits (Johnstone et al., 2013); blackcap (*Sylvia atricapilla*) (Leniowski and Węgrzyn, 2018); zebra finches (Mariette and Griffith, 2012); dovekie (*Alle alle*) (Wojczulanis-Jakubas et al., 2018); long tailed tits (*Aegithalos caudatus*) (van Rooij and Griffith, 2013); rock sparrow (*Petronia petronia*) (Baldan and Griggio, 2019)]. As with the coordination of incubation bouts, the coordination of nestling provisioning seems to be an active process, and there are many potential fitness advantages of synchronized provisioning, such as decreasing risk of predation during active foraging and minimizing trips to the nest. Indeed, in great tits, it has been shown that parents may adjust their provisioning of chicks more in response to their partner than to the chicks’ begging calls (Hinde and Kilner, 2006).

There are several lines of evidence suggesting that partner coordination improves reproductive success for the pair (Mariette and Griffith, 2012, 2015), minimizes reproductive conflict (Baldan and Griggio, 2019), and can even decrease sibling conflict (Shen et al., 2010). One clear explanation for this fitness advantage is that coordinated provisioning trips decrease the total number of nesting disturbances and thus decreases predation (Bebbington and Hatchwell, 2015). Partners may also benefit from the increased vigilance of their partners (Elie et al., 2010; Mainwaring and Griffith, 2013). For example, in the rock sparrow (*Petronia petronia*), biparental care is highly variable and typically one parent (usually the male) will desert the brood at some point prior to the fledging of chicks. Pairs that do not desert, but remain together, appear to be more synchronized (Baldan and Griggio, 2019). More specifically, partners that remain together have higher levels of alternation of nest visits during provisioning of chicks and increased synchronization of visits (Baldan and Griggio, 2019). However, the benefit of parental coordination is species-specific.

### Physiological Mechanisms and Consequences of Behavioral Synchrony

There has been very little research investigating the physiological mechanisms underlying parental coordination or the reciprocal impact of parental coordination on an individual’s brain and behavior. There is, however, considerable evidence that the coordination of reproductive physiologies is important (pairs need to be reproductively ready to breed at the same time), and in species that actively maintain life-long pair bonds the synchronization of yearly patterns in circulating hormone levels within a pair is associated with reproductive success. In graylag geese (*Anser anser)* and domestic geese (*Anser anser domesticus*), yearly patterns of circulating testosterone levels are correlated within a pair, and pairs with more coordinated patterns of circulating testosterone have greater reproductive success (Hirschenhauser et al., 1999, 2010; Hirschenhauser, 2012). Hirschenhauser (2012) proposed several possible explanations for this relationship. Pairs with higher testosterone coordination may be more coordinated in their reproductive physiology, and subsequently in their expression of appropriate hormonally-mediated behaviors. Alternatively, hormonal coordination could be a reflection of how behaviorally and hormonally responsive an individual is to their mate (Hirschenhauser, 2012). Thus, hormonal synchrony may be a cause or consequence of reproductive success. Furthermore, it is unclear whether it is important for biparental care or pair bonding *per se* (Hirschenhauser, 2012).

In a few species, reproductive success has also been linked to pair-level similarity in circulating glucocorticoid levels, although the relationship between the similarity in partner’s glucocorticoid levels and reproductive success varies across species. In great tits, pairs with high reproductive success have similar baseline corticosterone levels (Ouyang et al., 2014); additionally, circulating corticosterone levels become more similar between pairs the longer they are together (Ouyang et al., 2014). Similarly, in barn owls (*Tyto alba*) reproductive success is higher for pairs that have greater similarity in baseline corticosterone levels during incubation, but a greater dissimilarity in stress-induced circulating corticosterone levels during provisioning of chicks (Béziers et al., 2019). However, for eastern bluebird (*Sialia sialis)* parents, similarity in hormone levels (within the pair) does not appear related to reproductive success; although, individual hormonal levels are related to the expression of male and female parental behavior (Burtka et al., 2016). Combined, even when hormonal similarity does relate to reproductive success, it is unclear how such hormonal alignment would afford pairs greater reproductive success.

At this point it would be purely speculative to say whether relationships between parental coordination and hormonal synchrony are a cause or consequence of behavioral synchrony during brief interactions. One potential strategy that may allow us to disentangle behavioral synchrony (during brief interactions), parental coordination, and hormonal synchrony is to expose pairs to perturbations, disrupting levels of coordination between partners. In general behavioral disruptions are valuable in eliciting pair-directed behavior (Prior et al., 2014, 2016), and disrupting parental coordination has been effectively used to clarify the behavioral mechanisms underlying parental coordination (Boucaud et al., 2016a, b, 2017).

### Summary

Together these lines of work highlight the extent to which behavioral coordination between partners may be critical for biparental care in many species. The research presented above nicely operationalizes parental coordination over larger timescales. While many lines of evidence suggest that the benefit of parental coordination is a reduced predation risk, it is noteworthy that even in laboratory situations behavioral coordination between partners has been linked to improved reproductive success. For example, in the cockatiel (*Nymphicus hollandicus*), a gregarious Australian parrot, the coordination of activities prior to breeding was associated with improved fecundity during subsequent breeding periods (Spoon et al., 2006). Additionally, in the common marmoset (*Callithrix jacchus*), an individuals’ contribution to parental care is associated with hormonal synchrony and relationship quality (Finkenwirth et al., 2015; Finkenwirth and Burkart, 2017, 2018). The fact that parental coordination clearly affords a fitness advantage suggests there may be strong selection pressures supporting behavioral synchrony, although this could be consistent with the notion that coordination is more related to the sharing of parental duties than to monogamy.

Importantly while this section has focused on birds, numerous studies across a range of species highlight that behavioral compatibility generally improves reproductive success [e.g., convict cichlid *Amatitlania siquia* (Laubu et al., 2016); mound-building mouse, *Mus spicilegus* (Rangassamy et al., 2015)]. In prairie voles, it is social rather than genetic monogamy that has been linked to increased reproductive success (Ophir et al., 2008). However, it is also important to note that the coordination of parental duties is not unique to monogamous systems. Cooperatively-breeding species also actively coordinate nestling provisioning, form family-bonds, and display hormonal synchrony (Raihani et al., 2010; Finkenwirth et al., 2015; Finkenwirth and Burkart, 2017, 2018; Savage et al., 2017). In colonial species, there is evidence that reproductive synchrony can promote affiliative relationships even outside of the pair bond (Brandl et al., 2019). The interrelationship between patterns of sociality, parental care, parental coordination, and moment-to-moment behavioral synchrony could be disentangled by comparing closely related bird species across a range of social ecologies and mating systems.

What is particularly striking from the behavioral ecology work on birds is the extent to which brief social interactions actively coordinate parental coordination on longer timescales. In other words, these systems support the notion that behavioral coordination “scales up.” This is striking because behavioral synchronization does not require active communication and negotiation (Dostálková and Špinka, 2007). Many monogamous seabirds share incubation duties evenly (Black and Hulme, 1996) but there may be little room for such negotiation between partners in the timing of nest reliefs. In these species, the foraging partner waits to recover its body mass before returning to relieve the incubating partner, despite the fact that a delayed return may cause the incubating partner to abandon the nest (Davis, 1982; Chaurand and Weimerskirch, 1994; Yorio and Boersma, 1994). However, if we consider the number of bird species with nest-specific vocal displays [red-winged blackbirds (*Agelaius phoeniceus*) (Beletsky and Orians, 1985); white-throated dipper (*Cinclus cinclus*) (Villain et al., 2017); black-capped chickadees (*Poecile atricapillus*) (Otter et al., 2007); European robin (*Erithacus rubecula*) (Tobias and Seddon, 2002); yellow warbler (*Setophaga petechia*) (Moore and Rohwer, 2012)], it seems likely that active negotiation is a more widely spread phenomenon than we realize. Across bird species, there is tremendous potential to investigate the relationship between moment-to-moment behavioral synchrony and life-long patterns of coordination. However, in order to do so, we need to better operationalize behavioral synchrony and coordination during brief social interactions across species and contexts.

## Interpersonal Coordination in Humans

While behavioral ecology research has largely focused on coordination of activities and movements across hours and days, research from psychology, sociology and anthropology, on humans has had a much stronger focus on moment-to-moment behavioral synchrony during brief social interactions. Such research has focused considerable effort on operationalizing and disentangling concepts associated with the emergent and dynamic nature of behavioral synchrony, or “interpersonal coordination” as it is commonly termed in humans. In human research, the notion that interpersonal coordination is used (intentionally or not) to establish social bonds and connections has existed for over a century, at least since 1912 [(Durkheim, 1912) cited in Rennung and Göritz (2016)]. It has been recognized that the term coordination is difficult to define objectively. The term is used broadly and invokes the notion of harmonious working of multiple components (Bernieri et al., 1994), and carries connotations of cooperation, collaboration, and working-together. The challenge in operationalizing what is altogether obvious, yet surprisingly complex, is reflected in the wide range of specialized terms used to capture aspects of coordination and synchrony. There are over 15 terms used in the literature. In Table 1, I define, give examples and key references for, many of these specialized terms. Overall, “coordination” is commonly used to encompass many dynamic and emergent aspects of different features of human social interactions, whereas “synchrony” is a more specialized term that captures the temporal alignment of activities.

**TABLE 1:** Definitions of terms used to describe behavioral synchrony/interpersonal coordination in humans.

**Term**	**Definition**	**References**
Interpersonal coordination	During a social interaction, when the behaviors of individuals are patterned and synchronized; individuals displaying roughly the same behavior at the same time.	Lakin and Chartrand, 2003
	The coordination of postures and mannerisms between social partners.	Vicaria and Dickens, 2016
	Can be divided into behavioral mimicry and interactional synchrony.	Bernieri and Rosenthal, 1991; Rennung and Göritz, 2016
	Spontaneous temporal synchronization of body movements and/or speech between individuals in a social interaction.	Cornejo et al., 2017
	When behaviors in an interaction are non-random, patterned, or synchronized in both timing and form.	Bernieri and Rosenthal, 1991; Cornejo et al., 2017
Synchrony	To perform the same movement at the same time (*Synchronize).*	Tarr et al., 2016
	When two or more events happen at precisely the same time.	McDowall, 1978
	The coordination of movements between individuals in social interactions.	Bernieri et al., 1988
	Coordination of interpersonal behaviors.	Reddish et al., 2020
Interactional synchrony	The flow of movement in the listener is rhythmically coordinated to the flow of speech in the speaker.	Condon and Ogston, 1966; Condon and Ogston, 1967; Kendon, 1970
	When the boundaries of the movement of the listener coincide with the boundaries of the movement of the speaker. The listener and speaker may be making different movements.	Condon and Ogston, 1966; Condon and Ogston, 1967; Kendon, 1970
	The matching of rhythmic behaviors between individuals.	Reddish et al., 2020
	Precise speech-movement and movement-movement coordination between a speaker and listener.	McDowall, 1978
	Movement coordination during social interactions (syn with *interpersonal coordination).*	Bernieri et al., 1994
Intrapersonal synchrony	Synchronization of a person’s body movements to their speech rhythm.	Bernieri et al., 1988
Interpersonal synchrony	When the movements of two people overlap in time. However, interpersonal synchrony is not limited to behavioral synchrony, but includes synchrony on neural, physiological, and affective levels.	Rennung and Göritz, 2016
	The matching of rhythmic behaviors between individuals.	Reddish et al., 2020
	The matching of behavior in form and time.	Miles et al., 2010
	When an individual synchronizes their rhythm and movement with another person with whom they are interacting.	Bernieri et al., 1988
	The temporary alignment of periodic behaviors with another person.	Cacioppo et al., 2014
	Instances when two peoples’ movements are overlapping in time.	Rennung and Göritz, 2016
Behavioral synchrony	To perform the same action at the same time (*synchronous behavior*).	Dong et al., 2015
	Physically keeping together in time with others.	Baimel et al., 2018
Phase synchrony	*In-Phase Synchrony:* When the actions of each individual are simultaneously at equivalent points of the movement cycle (or a 0° relative phase relationship).	Kelso, 1995; Lumsden et al., 2012; Rennung and Göritz, 2016
	*Anti-Phase Synchrony:* When actions are simultaneously at opposite points of the cycle (or a 180° relative phase relationship).	Lumsden et al., 2012; Rennung and Göritz, 2016
Behavioral mimicry	When people engage in the same behavior (e.g., mannerisms, postures, motor movements) at the same time.	Chartrand and Lakin, 2013
	*Non-conscious behavioral mimicry:* The unwitting imitation of another’s behaviors.	Lakin and Chartrand, 2003
	*Non-conscious behavioral mimicry:* Instances in which individuals enact movements previously engaged in by others within the context of a social interaction.	Valdesolo and DeSteno, 2011
Biological rhythms	When one cyclical process is captured by and set to oscillate with another cyclical process.	Bernieri et al., 1988
Motor-sensory interpersonal synchrony (MSIS)	Referring to both the synchronization of motor movements and the synchronization of sensory stimulation.	Rennung and Göritz, 2016
Synchronous multisensory experiences	When individuals have a synchronous sensory experience (e.g., experimental manipulation of touch).	Paladino et al., 2010; Mazzurega et al., 2011; Rennung and Göritz, 2016
Movement synchrony	Non-verbal behavior of one person is highly interrelated, coordinated, attuned, aligned, or synchronized with the non-verbal behavior of their interaction partner.	Bernieri et al., 1994; Tunçgenç and Cohen, 2016; Altmann et al., 2019
Behavioral social synchrony	The coordination of behavior between two individuals (synonym social synchrony).	Kinreich et al., 2017
Emotional contagion	The automatic mimicry and synchronization of another’s vocalizations, postures, and movements.	Hatfield et al., 1993
	When a person reads, and spontaneously takes on the emotional and affective state of another.	Chartrand and Lakin, 2013
Behavioral entrainment	The adjustment or moderation of behavior to coordinate/synchronize with another.	Bernieri et al., 1988
Bio-behavioral synchrony	The coupling of individuals’ physiology and behavior during moments of social contact.	Feldman et al., 2011; Kinreich et al., 2017
	The coordination of physiological and behavioral processes among affiliated members during social contact.	Feldman, 2015
Brain-to-brain synchrony	Correlations in patterns of brain activity between people.	Hasson, 2016; Kinreich et al., 2017
	*Brain to brain coupling:* The perceptual system of one brain can be coupled to the motor system of another.	Hasson et al., 2012

*The terms presented here come from the fields of psychology, sociology and anthropology and are used to operationalize behavioral synchrony/interactional coordination. This is by no means an exhaustive list of terms used across these fields. The definitions provided in this table are taken with only the smallest changes possible to the wording of the given reference(s), to facilitate readability. From this range of terms, it is evident that there is considerable overlap across the social phenomena being described; however, the differences present across definitions reflect several key important features including: (1) the extent to which movements are temporally aligned and/or simultaneous, (2) the modality or domain of the behavior (and whether other biological or physiological processes are included), and (3) the intentionality underlying the behavioral interaction. It is also noteworthy that there are many instances where research is referenced using consistent terminology of the given manuscript (e.g., interpersonal synchrony), despite the fact that the original reference used a different term (e.g., interpersonal coordination). Additionally, many research articles use terms very generally with little or no definition provided. In particular, synchrony and coordination are often used without definition and/or in the definition of other terms.*

From the range of terms presented in Table 1, it is evident that there is considerable overlap across the social phenomena being described. However, these terms also capture key differences in dimensions or nuances of interpersonal coordination. The definitions reflect several key important features including, (1) the extent to which movements are temporally aligned and/or simultaneous, (2) the modality or domain of the behavior (and whether other biological or physiological processes are included), and (3) the intentionality underlying the behavioral interaction. However, both terms, synchrony and coordination, can also be used without precise definitions, apparently referring to the same overarching phenomena as well as in the definitions of more specialized terms. For consistency when discussing the range of research on human literature, I will use the term *interpersonal coordination*, [i.e., spontaneous coordination patterns between people during social interaction (Bernieri et al., 1988; Cornejo et al., 2017)] to refer broadly to coordination/synchrony. A key premise in research on human interpersonal coordination is that the social interactions themselves, and not the individuals separately, are typically the unit of analysis (Scheflen, 1982). Importantly, interpersonal coordination is not limited to behaviors alone, but includes physiological and behavioral coordination, and is associated with perception of social success in these moments.

Here, I do not exhaustively summarize the extensive bodies of work on human interpersonal coordination [see recent reviews and meta-analyses (Rennung and Göritz, 2016; Cornejo et al., 2017)]. Rather I aim to (1) provide evidence that humans are easily able to perceive and judge the degree of interpersonal coordination; (2) highlight the unified framework of bio-behavioral synchrony used in these bodies of work and emphasize the reciprocal relationship between physiology and interpersonal coordination; and (3) describe the consequences of interpersonal coordination for social relationships (romantic partnerships and pair bonds). Note that only for aim three will I restrict the discussion to references that have investigated interpersonal coordination between romantic couples, pair bonded individuals. Combined, this discussion is useful for elucidating the shared biological basis of features of synchrony and coordination across humans and non-human animals.

### Operationalizing Interpersonal Coordination

A breadth of methodologies have been used to capture and quantify interpersonal coordination in humans (reviewed in Cornejo et al., 2017). These methods range from micro-analysis of video recordings and motion tracking to various physiological measurements. Some of the earliest work was done by coding video recordings frame-by-frame (Condon and Ogston, 1966). Using this technique, researchers highlighted the precise temporal synchrony in movements that occur between speakers in a conversation even if they are not looking at each other (Kendon, 1970). This early work emphasized the role temporal synchrony plays in marking who is participating in a conversation and thus its crucial role in social interaction across contexts (Kendon, 1970).

Over the past several decades there have been many advances in technologies beyond frame-by-frame coding of videos. Now there are several different approaches for automatically scoring temporal synchrony in video recordings. Automatic detection methods have been employed for two decades (Grammer et al., 1999), and continue to be used. These methods have been used across a wide range of social contexts: including to describe the relationship between interpersonal coordination and patient satisfaction in doctor-patient interactions (Ramseyer and Tschacher, 2011), as well as to describe how interpersonal coordination is diminished when two people are arguing (Paxton and Dale, 2013). Movement synchrony has also been captured using motion sensors, such as accelerometers, potentiometers, electrogoniometers, magnetic motion capture systems, and optical motion capture systems. Again, these methods are commonly used today and have been applied to many contexts. Such quantifications of behavioral synchrony or interpersonal coordination have also been linked to psychological factors (e.g., perceived self-other merging, entitativity, liking, and trust) (Paladino et al., 2010; Mazzurega et al., 2011; Rennung and Göritz, 2016; Vicaria and Dickens, 2016). Importantly these behavioral measures are correlated with subjective feelings of synchrony, or connectedness between individuals (Llobera et al., 2016; Preissmann et al., 2016).

Interpersonal coordination, as captured by a range of these above described methods, has been linked to a wide range of physiological measures, including respiration, heart rate, and galvanic skin response to patterns of brain activity. This has been termed bio-behavioral synchrony, which conceptualizes the interrelationship between behavioral and physiological measures of synchrony (Feldman, 2012a). Romantic partners, for example, have been shown to synchronize across these measures: respiration (Helm et al., 2012); heart rate (Levenson and Gottman, 1983); galvanic skin response (Chatel-Goldman et al., 2014); and brain activity (Kinreich et al., 2017). Combined, these varied methods have emphasized how integrated the phenomenon is across behavioral-physiological markers. Furthermore, they highlight the wide range of entry points to study interpersonal coordination across contexts.

### Are We in Sync? The Perception of Interpersonal Coordination

Early research on interpersonal coordination cautiously made the assumption that aspects of synchrony were beyond human perception (Condon and Ogston, 1967). Some of the earliest work developed objective criteria for scoring synchrony and experimentally demonstrated that interactional synchrony could be consistently rated by untrained observers (Bernieri et al., 1988). In these early experiments, the only guidance observers were given were brief instructions on how to score three aspects of synchrony:

(1) **Simultaneous movement** – this reflects the quantity or degree of movement that appears to begin or end at the same moment. For example, if a mother begins to turn her head at the precise moment that a child lifts an arm off of a table, it is an instance of simultaneous movement.
(2) **Tempo similarity** – assume that all people have built-in tempos or speeds at which their behavior is set (much like the tempo an orchestra follows at a concert). Rate the degree to which the two people in the clip appear to be “marching to the beat of the same drummer.”
(3) **Coordination and smoothness** – assume you are viewing a choreographed dance instead of a social interaction. How smoothly does the interactants’ flow or behavior intertwine, or mesh evenly and smoothly?
  *As given in the rating* from page 246 (Bernieri et al., 1988).

Asked to rate social interactions on a scale of 0–9 for each of these three features of synchrony, observers were able to rate social interactions consistently, and expected patterns emerged: for example, mother-infant interactions were rated as being more in sync than those mothers with an infant that was not their own (Bernieri et al., 1988). Untrained observers were also able to rate the degree of interpersonal coordination when only shown the gross features of an interaction (body movements) without seeing the fine details, such as facial expressions and small movements (twitches) (Bernieri et al., 1994). Observer or participant ratings of interpersonal coordination are still commonly used today to investigate the role and impact of interpersonal coordination on a variety of social conditions (Cacioppo et al., 2014; Koehne et al., 2016; Llobera et al., 2016; Preissmann et al., 2016; Koudenburg et al., 2017). Importantly, a recognition of the salient features of interpersonal coordination has led to discoveries that interpersonal coordination can have positive behavioral outcomes even for those not involved in the interaction directly (reviewed in Vicaria and Dickens, 2016).

### Behavioral and Physiological Levels of Interpersonal Coordination

The interconnectedness of behavioral and physiological synchrony has been recognized for a long time, and such theories have been well developed, largely coming from early research on mother-infant attachments (Feldman, 2007, 2012a,b, 2015). For mother-offspring relationships, the role of coordination of behavior and physiology on the developing affiliative bond is particularly striking. Through repeated social interactions, parents and offspring become increasingly responsive or sensitized to the physiological and behavioral cues of the other, forming an integrated mother-offspring unit that displays increasing synchronization and forges a selective and enduring attachment (Fleming et al., 1999; Feldman, 2012a). The theory of *bio-behavioral synchrony* has been used to describe these embodied phenomena. During repeated social interactions, individuals’ physiological responses such as heart rhythms, endocrine state, and brain activity become correlated and are shaped by the presence of the emerging parent-offspring bonds (Feldman, 2007, 2012a,b, 2015).

### Interpersonal Coordination, Prosocial Behavior and Pair Bonding

It has long been known that humans are exquisitely good at synchronizing behaviors during brief social interactions. Adults are capable of behaviorally aligning with any conspecifics including family, romantic partners, friends (familiar conspecifics), and strangers (Feldman, 2012a, b, 2015; Ulmer-Yaniv et al., 2016). This is not surprising since humans are extremely social and maintain many affiliative bonds of varying degrees and types. Interpersonal coordination has been well-described across many types of social dyads including mother-infant, parent-child, doctor-patient, teacher-student, romantic partners, and strangers (Rennung and Göritz, 2016; Cornejo et al., 2017).

Across contexts, interpersonal coordination has been shown to signal interest and positive affect (Bernieri and Rosenthal, 1991); facilitate cooperation (Wiltermuth and Heath, 2009); reflect the relationship of the social partners (Kinreich et al., 2017) and rapport (Lakin and Chartrand, 2003); promote prosocial behavior; and galvanize members of a group to collaborate on tasks (Mu et al., 2017). A recent meta-analysis (Rennung and Göritz, 2016) summarized the results of 60 experimental studies that investigated potential functions of interpersonal coordination. Here the authors distinguish between both motor interpersonal synchrony (when individuals move in sync) and sensory interpersonal synchrony (when individuals receive a sensory stimulation at the same time). The 60 experimental studies either examined the effect of interpersonal synchrony on prosocial attitudes (i.e., perceived self-other merging, entitativity, unity, closeness, similarity, liking, and trust) and/or on prosocial behavior (i.e., cooperation, conformity, helping behavior, and other-related attention such as social memory). Across these studies, there is strong evidence that interpersonal coordination enhances both prosocial attitudes and behaviors. Importantly, the consequences of interpersonal coordination on prosocial attitudes does not appear to depend on whether the synchronization was intentional; although, intentionality may enhance the effect of synchrony on expressions of prosocial behavior (Rennung and Göritz, 2016). Such a general relationship between synchrony and prosociality may emphasize the role of interpersonal coordination in social bonding very generally. This also raises the question of how the behavioral-physiological process involved in interpersonal coordination between strangers is similar to those between romantic partners.

As with other affiliative relationships in humans, romantic partnerships (pair bonds) are characterized by interpersonal coordination. Similar to the formation and maintenance of parent-offspring relationships, the formation and maintenance of romantic partnerships is also characterized by a concordance in behavioral and physiological synchrony (biobehavioral synchrony) (Schneiderman et al., 2012; Scheele et al., 2013; Ulmer-Yaniv et al., 2016; Kinreich et al., 2017; Sharon-David et al., 2018). Interestingly, some of these lines of evidence for biobehavioral synchrony between romantic partners comes from changes that occur in early parents. For example, in the first year of parenting, first time mothers and fathers develop correlations between circulating endocrine levels (oxytocin) (Feldman et al., 2007), and such hormonal synchrony between mothers and fathers is predictive of family level behavioral synchrony (Gordon et al., 2010). These phenomena parallel some of the patterns described with behavioral and hormonal synchrony in biparental birds, and raise similar questions about our ability to disentangle the coordination of shared parental duties from other aspects of a partnership.

Another useful comparison to understand the effect of romantic partnerships on interpersonal coordination comes from comparisons between romantic partners and strangers. Importantly, small differences in key aspects of micro-social interactions can have profound differences on physiological indicators of synchrony. Kinreich et al. (2017) used hyperscanning EEG to investigate the connections between behavioral and neural synchrony (brain-to-brain coupling). They show that neural synchrony between couples is unique to periods of social interactions (i.e., is not present at rest) and is related to non-verbal cues between couples rather than speech and features of conversations (Kinreich et al., 2017). Couples and strangers did not differ in their overall affect (amount of time spent in positive affect) nor in topics of conversation or amount of time speaking. However, couples spent more time making eye contact, and neural synchrony was higher specifically during these periods of shared gaze. For strangers, neural synchrony was not elevated during periods of shared gaze, however, there was a correlation across dyads in the amount of social gaze and neural synchrony.

### Summary

Extensive bodies of work have described the role of interpersonal coordination in human social connections and relationships. Here I have highlighted (1) some of the complexities and nuances that exist in operationalizing, defining and scoring aspects of coordination, (2) the awareness humans have of the extent to which dyads are synchronized, (3) the pervasiveness of synchrony, not only as a behavioral expression, but also as a behavioral-physiological phenomenon at the level of a dyad or group, and finally (4) the effect of pair bonding (and social bonding more broadly) on interpersonal synchrony. For this last point, it is particularly important to note that during very brief social interactions subtle behavioral exchanges can have striking impacts on human connectedness. Again, I want to emphasize that while brain-to-brain coupling in humans is linked to pair bonding, this type of synchrony is not simply an intrinsic response to being bonded, rather it is developed and cultivated over time through repeated social interactions and can also be achieved through other mechanisms such as shared memory or immediate responses to narratives (Hasson, 2016; Chen et al., 2017; Liu et al., 2017; Mu et al., 2017).

## A Case Study: Assessing Behavioral Synchrony During Brief Social Interactions in Zebra Finch Dyads

Above I have discussed the extensive bodies of research from behavioral ecology highlighting the manner in which parental behavior is actively negotiated during brief periods of social interactions, suggesting that moment-to-moment behavioral synchrony may be a key aspect of monogamous partnerships. Furthermore, research from human psychology offers in-depth descriptions of how to conceptualize and operationalize moment-to-moment interactional synchrony and provides robust experimental evidence that behavioral synchrony during brief interactions is key to developing social connections and social bonds. Combined, the above two bodies of work raise the question of how moment-to-moment behavioral synchrony during brief social interactions is related to social bonding in birds and other animals.

Now I turn to some of my recent research, in zebra finches, where I quantify multimodal patterns of behavioral synchrony during brief greets (or reunions). My aim was to describe how pair bonding influenced patterns of behavioral synchrony outside of a breeding context. Greeting (reunion behavior) represents a social situation that is relevant as pair bonds mature (over time, and across breeding stages), as well as across social dyads (with pair bonded mates as well as other flock mates). Ultimately, greeting behavior may provide a relevant social scenario that could be compared across species. The two experiments I discuss below describe how behavioral synchrony (1) is affected by time (over the early stages of pair bonding), and (2) differs across social relationships.

### The Ecology and Ethology of Zebra Finches

Zebra finches typically form life-long sexually monogamous pair bonds, but are also socially tolerant and breed and travel colonially (Birkhead et al., 1990; Zann, 1996). They engage in biparental care, and the male and female divide parental duties relatively evenly. Furthermore, zebra finches breed opportunistically and thus make breeding decisions at the level of the pair after integrating multiple social and environmental cues (Perfito et al., 2007; Prior et al., 2013; Prior and Soma, 2015), making the need to coordinate behaviors and reproductive bouts particularly important. Finally, zebra finches have a large repertoire of affiliative behaviors, including dynamic calling behavior, which are used with their monogamous partner as well as other familiar conspecifics (Zann, 1996; Elie et al., 2010, 2011a,b).

Zebra finch pairs do not hold and defend territories, and they remain gregarious. Interestingly, in the absence of an opposite-sex partner they will form equally strong social bonds with same-sex conspecifics, and in the laboratory it appears individuals can also maintain multiple social bonds (Alger et al., 2011; Elie et al., 2011a; Tomaszycki and Zatirka, 2014). Because zebra finches are gregarious, they do not show the increased aggression toward novel opposite-sex individuals that marks the establishment of a pair bond in rodents as described in the introduction. Furthermore, traditional partner preference paradigms may not show selective preference for partners (Prior et al., 2013), although other behavioral assays clearly show that the monogamous bond is selective (Gill et al., 2015; Fernandez et al., 2017).

### Assessing Multimodal Behavioral Synchrony in Zebra Finches

**FIGURE 1:**
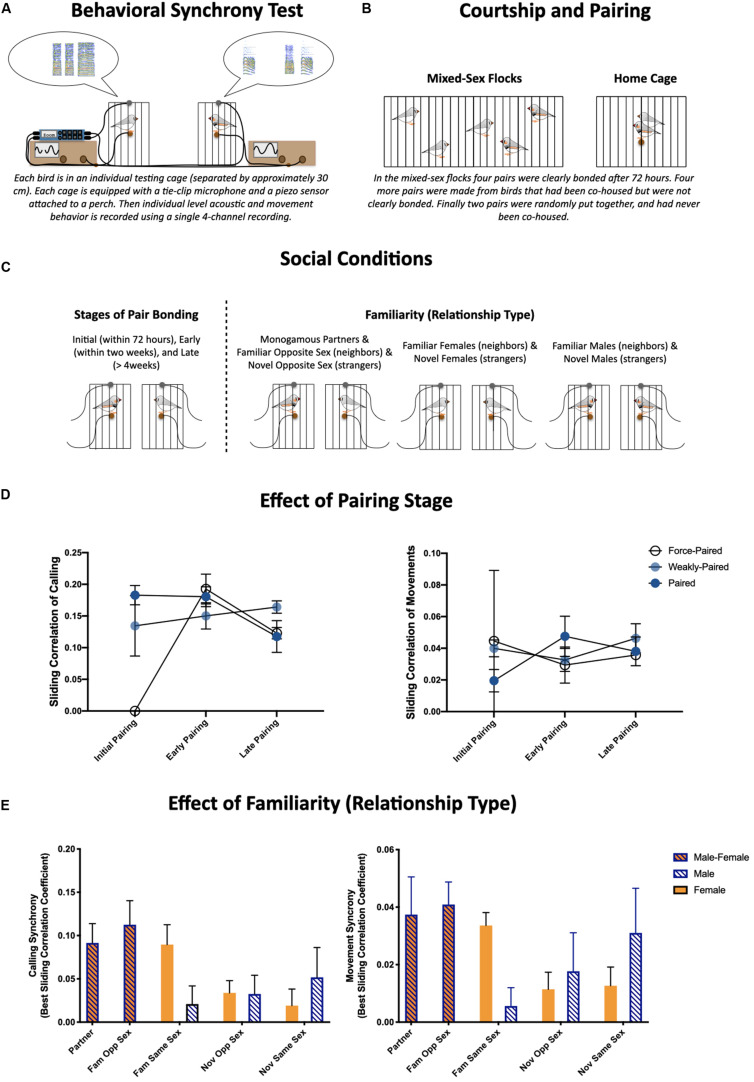
**(A)** Schematic of the behavioral synchrony paradigm used to quantify behavior during social reunions (greets). The finer, moment-to-moment details of these interactions were quantified from single four-channel recordings of acoustic data from tie-clip microphones and movement data from piezo sensors attached to each perch. **(B)** Illustration of paradigm used to set up pairs. **(C)** We quantified behavioral synchrony during social reunions over the course of pair bonding [initial pairing (4–72 hours), early pairing (within the first two weeks), and late pairing (>4 weeks) (Left)], and across different social relationships (Monogamous partners, familiar same- and opposite-sex dyads as well as novel same- and opposite-sex dyads). **(D)** Effects of pairing stage and prior experience on behavioral synchrony (temporal synchrony based on sliding correlation coefficients) of calls (left) and movements (right) (Prior et al., 2020). **(E)** Effects of social relationship on behavioral synchrony (temporal synchrony based on sliding correlation coefficients) of calls (left) and movements (right) (Prior et al., 2019).

Given both the importance of behavioral coordination for monogamous partners during biparental care and the implication of behavioral synchrony broadly in supporting formation and maintenance of social relationships, one might predict that moment-to-moment behavioral synchrony is heavily affected by pair bonding. Furthermore, it would be natural to predict that behavioral synchrony is higher between monogamously bonded individuals than other social dyads. We tested these hypotheses by quantifying multimodal behavioral synchrony during brief social interactions (reunions or greets) (Figure 1A) in zebra finch dyads across social conditions (Prior et al., 2019, 2020) (Figures 1B,C).

Similar partner separation and reunion paradigms have been used in zebra finches previously (Prior et al., 2014, 2018). We focused on the first 5 min of behavior during interactions or brief reunions following a short (about 3 min) separation or disruption. The finer, moment-to-moment details of these interactions were quantified by recording acoustic data from a tie-clip microphone and movement data from a piezo sensor attached to the perch of a smaller cage along with audio recordings using a single multi-channel Zoom recorder (F8) (Figure 1A). Pairs were allowed to freely form in mixed-sex flocks for 72 h (Figure 1B). Pair bonding was assessed visually each day: occurrences of selective affiliative behaviors (i.e., clumping, allopreening, and coordinated preening) were scored between individuals during 5 min behavioral observations. Four pairs clearly formed bonds during this time (paired), another four pairs were created from these flocks who were not strongly affiliative (weakly-paired), and two pairs were formed across flocks who had no prior experience with each other (force-paired).

As described throughout this review, there are many ways to quantify the coordination or synchronization of behavior. For these experiments we (1) quantified the similarity in activity levels between individuals within a dyad, (2) calculated sliding correlation coefficients of time-stamped calls (and movements) as a quantification of the temporal synchronization within a dyad, and (3) conducted principal component analyses on activity levels and sliding correlations coefficients (for calls and movements) to describe multimodal behavioral patterns.

With respect to the first hypothesis, we showed calling activity during greeting behavior was highest during initial courtship, and there was a general pattern of decreased activity across the three stages of pair bonding (initial, early, and late pairing) (Figure 1C). Despite differences in activity levels, the coordination of activities remained largely constant, however, the two pairs that were force-paired prior to the courtship recording were much less coordinated during the courtship phase (sliding correlation coefficient for calls and movements is shown in Figure 1D) (Prior et al., 2020).

With respect to our second hypothesis, we found greeting behavior was affected by social relationship. Familiarity, particularly with females, resulted in more robust and more coordinated greeting behavior. More specifically, monogamous partners, familiar opposite sex dyads, and female familiar same sex dyads were more coordinated in both calling and movement, compared to novel dyads and familiar same sex male dyads (sliding correlation coefficient for calls and movements is shown in Figure 1E; Prior et al., 2019). It is also notable that we consistently found females were more active than males, both with respect to call and movement rate (Prior et al., 2019, 2020). These two results are consistent with each other in highlighting that prior social experience rather than pair bonding *per se* modulates moment-to-moment behavioral synchrony.

### Summary

These experiments are an early step toward describing multimodal patterns of behavioral synchrony in mundane social interactions across social contexts. The results of the two experiments are consistent in that they suggest that behavioral synchrony is not necessarily enhanced between monogamous partners; but is heavily influenced by prior social experience. Overall, these findings are consistent with the patterns described throughout the review and suggest that behavioral synchrony plays a general role in social relationships rather than being specific to pair bonding. The research described above on behavioral coordination in biparental birds suggests that parental coordination may be more related to sharing parental care rather than being an artifact of pair bonding. Additionally, the research described above on interpersonal coordination in humans would be consistent with the notion that higher behavioral synchrony in romantic couples is due to shared experiences rather than an intrinsic consequence of the formation of the partnership.

Combined, these areas of research all point to the importance of shared experience. They suggest that research exploring the shared biological foundations of social alignment may provide a rich basis for comparative studies that investigate the functions of behavioral coordination across timescales, species, and contexts. Such investigations would no doubt require longitudinal studies relating interpersonal coordination (or multimodal behavioral synchrony) over time to other measures of behavioral and physiological synchrony between pairs. Here again, experimental approaches that quantify the effects of a disruption to partner coordination at one level (e.g., parental coordination) on other levels of partner coordination (e.g., hormonal or parental coordination) (Boucaud et al., 2016a, b, 2017) would be important for disentangling different measures and consequences of behavioral synchrony across timescales.

## General Discussion and Future Directions

There are many challenges when it comes to expanding our understanding of pair bonding to incorporate the diversity that exists in pair bonding phenotypes. These challenges are multifaceted, but include: (1) many of the highly marked behavioral variables used are not applicable across contexts and species, (2) the ultimate functions as well as the behavioral and physiological mechanisms underlying pair bonding are confounded with biparental care, and (3) not all affiliative behaviors are equally important. Among a number of possible approaches, one general solution to all three of these challenges is identifying behavioral variables that are relevant across species and contexts. Here I propose using behavioral synchrony as a fundamental aspect of broader sociality, through which we can gain a deeper understanding of the diversity of pair bonding phenotypes across species and contexts.

Comparing patterns of behavioral synchrony in marked interactions of pair-bonded individuals (e.g., monogamous displays, courtship behavior, parental behavior) to behavioral synchrony in general social interactions (such as greetings) could offer a more detailed, nuanced portrait of the dynamic processes of social alignment. The patterns described in this review, including the role of active negotiation during brief social interactions on parental coordination, as well as the impacts of interactional synchrony on brain-to-brain coupling, suggest that behavioral coordination is seen across timescales and physiological levels. Thus, research on behavioral synchrony may prove invaluable for developing an understanding of how pair bonds change over time and are affected by social and environmental conditions. However, before comparing behavioral synchrony across contexts and species, further work needs to be conducted to determine how synchrony is related across behavioral-physiological levels. Importantly, such research needs to put synchrony within the context of pair bonding, and control for potential confounds that come from relating pair coordination to reproductive behavior. Such research lines will contribute to our understanding of whether moment-to-moment behavioral synchrony provides a basis for larger-scale behavioral alignment or vice versa.

When considering the interrelatedness of synchrony across behavioral-physiological levels, it is important to acknowledge that research on the neurobiology of behavioral synchrony is organized very differently than the research identifying neural circuits associated with social bonding. These two bodies of work offer very different perspectives on the neurobiological underpinnings of complex social dynamics. Research on the neurobiology of pair bonding has focused on identifying the key “players”: the brain regions, circuits, and neuromodulators that are implicated in the formation of a monogamous bond (Aragona et al., 2006; Alger et al., 2011; O’Connell and Hofmann, 2012; Donaldson and Young, 2016). Neurobiology research on behavioral synchrony, on the other hand, has focused on relating behavioral synchrony to neural synchrony. For example, in the plain-tailed wren (*Pheugopedius euophrys*), neural recordings have demonstrated that the partners’ synchronized vocal duet is associated with tight correlation in the partners’ neural responses in a cortical brain region associated with vocal-motor integration (Fortune et al., 2011; Coleman and Fortune, 2018). It is particularly remarkable to note that the synchrony of neural firing between mates occurs in response to the entire duet (both female and male components) as a whole, not to each individual component alone (Fortune et al., 2011). It may be that combining these different perspectives on the neurobiology of social dynamics will prove valuable in expanding our understanding of the neurobiology of diversity in pair bond phenotypes. For example, recent research investigating the neurobiology of long-term pair maintenance in prairie voles has benefited from a similarly nuanced approach examining the consequences of pair bonding on brain and behavior (Scribner et al., 2019). These approaches could be combined by studying the consequences of behavioral synchrony or dis-synchrony on neural circuits associated with pair bonding, and reciprocally by identifying the role of pair bonding on neural synchrony between individuals in a dyad.

At the beginning of this review, I suggested that it is easy to assume that behavioral synchrony is positively related to, and perhaps qualitatively unique in, monogamous partnerships. However, throughout this review i have emphasized that behavioral synchrony is critical for all types of social bonds. It is possible that there is something unique about how behavioral synchrony interacts with pair bonding. If there are unique characteristics of behavioral synchrony in pair bonded individuals, it is likely more nuanced than simply the degree of synchrony. For example, it is possible that monogamously bonded pairs more easily regain synchrony following a long-term separation, and/or that the consequences of disruptions to synchrony between partners are greater than disruptions to synchrony between non-bonded individuals. Again, detailed descriptions of brief social interactions and precise quantification and operationalization of behavioral synchrony are needed to determine whether there are in fact unique relationships between behavioral synchrony and pair bonding.

Alternatively, it is also possible that there is nothing specialized about behavioral synchrony during pair bonding. Perhaps what makes pair bonds unique is simply the cumulation of unique shared experiences associated with courtship, biparental care, and long-term coordination of activities and movements. If behavioral synchrony is indeed not specialized in monogamous partnerships, this would argue for a shift in how we conceptualize monogamous partnerships: away from seeing them as extreme and unique social bonds, and toward recognizing that they exist along a continuum of varied social relationships.

Combined, these data suggest that moment-to-moment behavioral synchrony is easily perceptible (Bernieri et al., 1988) and information rich (Elie et al., 2010; Boucaud et al., 2016a, 2017; Villain et al., 2016), and that, even outside of breeding periods, it may promote reproductive success, a traditional metric of pair bond success (Spoon et al., 2006). The extent to which these patterns of synchrony hold within and across contexts and species remains to be tested. However, the existence of such a pattern suggests that regardless of whether behavioral synchrony is somehow specialized to monogamy, behavioral synchrony itself could be a metric of successful pair bonds. Various disruptions to behavioral synchrony could be assessed for their consequences on reproductive success, effects on frequency of extra-pair mating, effects on maintenance of other strong social bonds, and likelihood to divorce.

Altogether, there is overwhelming evidence that “moments matter,” and that even brief social interactions can have profound effects on monogamous partnerships. The significance of this conceptual framework is a recognition that pair bonds, as well as of other affiliative bonds, are built upon *repeated* social interactions and experiences and that bonds are co-created in the interactions between individuals, making them intrinsically emergent and dynamic in nature.

## Author Contributions

The author confirms being the sole contributor of this work and has approved it for publication.

## Conflict of Interest

The authors declare that the research was conducted in the absence of any commercial or financial relationships that could be construed as a potential conflict of interest.

## References

[B1] Alger S. J. Juang C. Riters L. V. (2011). Social affiliation relates to tyrosine hydroxylase immunolabeling in male and female zebra finches (*Taeniopygia guttata*). *J. Chem. Neuroanat.* 42 45–55. 10.1016/j.jchemneu.2011.05.005 21605658 PMC3148347

[B2] Altmann U. Schoenherr D. Paulick J. Deisenhofer A.-K. Schwartz B. Rubel J. A. (2019). Associations between movement synchrony and outcome in patients with social anxiety disorder: evidence for treatment specific effects. *Psychother. Res.* 30 574–590. 10.1080/10503307.2019.1630779 31213149

[B3] Andrew DeWoody J. Fletcher D. E. David Wilkins S. Nelson W. S. Avise J. C. (2000). Genetic monogamy and biparental care in an externally fertilizing fish, the largemouth bass (*Micropterus salmoides*). *Proc. R. Soc. Lon. Ser. BBiol. Sci.* 267 2431–2437. 10.1098/rspb.2000.1302 11133034 PMC1690830

[B4] Aragona B. J. Liu Y. Yu Y. J. Curtis J. T. Detwiler J. M. Insel T. R. (2006). Nucleus accumbens dopamine differentially mediates the formation and maintenance of monogamous pair bonds. *Nat. Neurosci.* 9 133–139. 10.1038/nn1613 16327783

[B5] Ashton–James C. Baaren R.B. Van Chartrand T. L. Decety J. Karremans J. (2007). Mimicry and me: the impact of mimicry on self–construal. *Soc. Cogn.* 25 518–535. 10.1521/soco.2007.25.4.518

[B6] Bailey I. Myatt J. P. Wilson A. M. (2013). Group hunting within the Carnivora: physiological, cognitive and environmental influences on strategy and cooperation. *Behavi. Ecol. Sociobiol.* 67 1–17. 10.1007/s00265-012-1423-3

[B7] Baimel A. Birch S. A. J. Norenzayan A. (2018). Coordinating bodies and minds: behavioral synchrony fosters mentalizing. *J. Exp. Soc. Psychol.* 74 281–290. 10.1016/j.jesp.2017.10.008

[B8] Baldan D. Griggio M. (2019). Pair coordination is related to later brood desertion in a provisioning songbird. *Anim. Behav.* 156 147–152. 10.1016/j.anbehav.2019.08.002

[B9] Ball G. F. Silver R. (1983). Timing of incubation bouts by ring doves (*Streptopelia risoria*). *J. Comp. Psychol.* 97 213–225. 10.1037/0735-7036.97.3.213 6684527

[B10] Bebbington K. Hatchwell B. J. (2015). Coordinated parental provisioning is related to feeding rate and reproductive success in a songbird. *Behav. Ecol.* 27 652–659. 10.1093/beheco/arv198 27193460

[B11] Beery A. K. Christensen J. D. Lee N. Blandino K. (2018). Specificity in sociality: mice and prairie voles exhibit different patterns of peer affiliation. *Front. Behav. Neurosci.* 12:50. 10.3389/fnbeh.2018.00050 29615879 PMC5868120

[B12] Beery A. K. Loo T. J. Zucker I. (2008). Day length and estradiol affect same-sex affiliative behavior in the female meadow vole. *Horm. Behav.* 54 153–159. 10.1016/j.yhbeh.2008.02.007 18387611 PMC2501115

[B13] Beery A. K. Routman D. M. Zucker I. (2009). Same-sex social behavior in meadow voles: multiple and rapid formation of attachments. *Physiol. Behav.* 97 52–57. 10.1016/j.physbeh.2009.01.020 19419672 PMC2798739

[B14] Beletsky L. D. Orians G. H. (1985). Nest-associated vocalizations of female red-winged blackbirds, *Agelaius phoeniceus*. *Z. Tierpsychol.* 69 329–339. 10.1111/j.1439-0310.1985.tb00156.x

[B15] Bernieri F. J. Davis J. M. Rosenthal R. Knee C. R. (1994). Interactional synchrony and rapport: measuring synchrony in displays devoid of sound and facial affect. *Personal. Soc. Psychol. Bull.* 20 303–311. 10.1177/0146167294203008

[B16] Bernieri F. J. Reznick J. S. Rosenthal R. (1988). Synchrony, pseudosynchrony, and dissynchrony: measuring the entrainment process in mother-infant interactions. *J. Personal. Soc. Psychol.* 54 243–253. 10.1037/0022-3514.54.2.243

[B17] Bernieri F. J. Rosenthal R. (1991). “Interpersonal coordination: behavior matching and interactional synchrony,” in *Studies in Emotion & Social Interaction. Fundamentals of Nonverbal Behavior*, eds Feldman R. S. Rimé B. (New York, NY: Cambridge University Press), 401–432.

[B18] Béziers P. Jenni L. Roulin A. Almasi B. (2019). Reproductive success in the barn owl is linked to partner compatibility in glucocorticoid levels. *BioRxiv* [Preprint]. 10.1101/517227

[B19] Birkhead T. R. Burke T. Zann R. Hunter F. M. Krupa A. P. (1990). Extra-pair paternity and intraspecific brood parasitism in wild zebra finches *Taeniopygia guttata*, revealed by DNA fingerprinting. *Behav. Ecol. Sociobiol.* 27 315–324. 10.1007/bf00164002

[B20] Black J. M. Hulme M. (1996). *Partnerships in Birds: The Study of Monogamy.* Oxford: Oxford University Press.

[B21] Boinski S. Garber P. A. (2000). *On the Move: How and Why Animals Travel in Groups.* Chicago, IL: University of Chicago Press.

[B22] Boucaud I. C. A. Mariette M. Villain A. Vignal C. (2016a). Vocal negotiation over parental care? Partners adjust their time spent incubating based on their acoustic communication at the nest. *Biol. J. Linnean Soc.* 117 322–336. 10.1111/bij.12705

[B23] Boucaud I. C. A. Smith M. L. N. A. Valère P. A. Vignal C. (2016b). Incubating females signal their needs during intrapair vocal communication at the nest: a feeding experiment in great tits. *Anim. Behav.* 122 77–86. 10.1016/j.anbehav.2016.09.021

[B24] Boucaud I. C. A. Valère P. A. Aguirre Smith M. L. N. Doligez B. Cauchard L. Rybak F. (2016c). Interactive vocal communication at the nest by parent Great Tits *Parus major*. *Ibis* 158 630–644. 10.1111/ibi.12374

[B25] Boucaud I. C. A. Perez E. C. Ramos L. S. Griffith S. C. Vignal C. (2017). Acoustic communication in zebra finches signals when mates will take turns with parental duties. *Behav. Ecol.* 28 645–656. 10.1093/beheco/arw189 27193460

[B26] Brandl H. B. Griffith S. C. Farine D. R. Schuett W. (2019). Wild zebra finches that nest synchronously have long-term stable social ties. *J. Anim. Ecol.* 10.1111/1365-2656.13082 31407336

[B27] Bull C. M. (2000). Monogamy in lizards. *Behav. Process.* 51 7–20. 10.1016/s0376-6357(00)00115-7 11074308

[B28] Bulla M. Valcu M. Rutten A. L. Kempenaers B. (2013). Biparental incubation patterns in a high-Arctic breeding shorebird: how do pairs divide their duties? *Behav. Ecol.* 25 152–164. 10.1093/beheco/art098 24347997 PMC3860833

[B29] Burtka J. L. Lovern M. B. Grindstaff J. L. (2016). Baseline hormone levels are linked to reproductive success but not parental care behaviors. *Gen. Comp. Endocrinol.* 229 92–99. 10.1016/j.ygcen.2016.03.010 26972151

[B30] Cacioppo S. Zhou H. Monteleone G. Majka E. A. Quinn K. A. Ball A. B. (2014). You are in sync with me: neural correlates of interpersonal synchrony with a partner. *Neuroscience* 277 842–858. 10.1016/j.neuroscience.2014.07.051 25088911

[B31] Carter C. S. (1998). Neuroendocrine perspectives on social attachment and love. *Psychoneuroendocrinology* 23 779–818. 10.1016/s0306-4530(98)00055-9 9924738

[B32] Chartrand T. L. Lakin J. L. (2013). The antecedents and consequences of human behavioral mimicry. *Annu. Rev. Psychol.* 64 285–308. 10.1146/annurev-psych-113011-143754 23020640

[B33] Chatel-Goldman J. Congedo M. Jutten C. Schwartz J.-L. (2014). Touch increases autonomic coupling between romantic partners. *Front. Behav. Neurosci.* 8:95. 10.3389/fnbeh.2014.00095 24734009 PMC3973922

[B34] Chaurand T. Weimerskirch H. (1994). Incubation routine, body mass regulation and egg neglect in the blue petrel *Halobaena caerulea*. *Ibis* 136 285–290. 10.1111/j.1474-919x.1994.tb01097.x

[B35] Chen J. Leong Y. C. Honey C. J. Yong C. H. Norman K. A. Hasson U. (2017). Shared memories reveal shared structure in neural activity across individuals. *Nat. Neurosci.* 20 115–125. 10.1038/nn.4450 27918531 PMC5191958

[B36] Clutton-Brock T. H. (1991). *The Evolution of Parental Care.* Princeton, NJ: Princeton University Press.

[B37] Cockburn A. (2006). Prevalence of different modes of parental care in birds. *Proc. R. Soci. B Biol. Sci.* 273 1375–1383. 10.1098/rspb.2005.3458 16777726 PMC1560291

[B38] Coleman M. Fortune E. (2018). Duet singing in plain-tailed wrens. *Curr. Biol.* 28 R643–R645. 10.1016/j.cub.2018.02.066 29870698

[B39] Condon W. S. Ogston W. D. (1966). Sound film analysis of normal and pathological behavior patterns. *J. Nervous Ment. Dis.* 143 338–347. 10.1097/00005053-196610000-00005 5958766

[B40] Condon W. S. Ogston W. D. (1967). A segmentation of behavior. *J. Psychiatr. Res.* 5 221–235. 10.1016/0022-3956(67)90004-0

[B41] Conradt L. Roper T. J. (2005). Consensus decision making in animals. *Trends Ecol. Evol.* 20 449–456. 10.1016/j.tree.2005.05.008 16701416

[B42] Cornejo C. Cuadros Z. Morales R. Paredes J. (2017). Interpersonal coordination: methods, achievements, and challenges. *Front. Psychol.* 8:1685. 10.3389/fpsyg.2017.01685 29021769 PMC5623900

[B43] Cramp S. Perrins C. M. (1982). *The Birds of the Western Palearctic: Flycatchers to Shrikes.* Oxford: Oxford University Press.

[B44] Davis L. S. (1982). Timing of nest relief and its effect on breeding success in Adelie penguins (*Pygoscelis adeliae*). *Condor* 84 178–183. 10.2307/1367665

[B45] Dewsbury D. A. (1988). “The comparative psychology of monogamy,” in *Comparative Perspectives in Modern Psychology, Proceedings of the Nebraska Symposium on Motivation*, Vol. 35 ed. Leger D. W. (Lincoln: University of Nebraska Press), 1–50. 3332030

[B46] Díaz-Muñoz S. L. Bales K. L. (2016). Monogamy” in primates: variability, trends, and synthesis: introduction to special issue on primate monogamy. *Am. J. Primatol.* 78 283–287. 10.1002/ajp.22463 26317875 PMC5474116

[B47] Donaldson Z. R. Young L. J. (2016). *The Neurobiology and Genetics of Affiliation and Social Bonding in Animal Models, Animal Models of Behavior Genetics.* Berlin: Springer, 101–134.

[B48] Dong P. Dai X. Wyer R. S. Jr. (2015). Actors conform, observers react: the effects of behavioral synchrony on conformity. *J. Personal. Soc. Psychol.* 108 60–75. 10.1037/pspi0000001 25437130

[B49] Dostálková I. Špinka M. (2007). Synchronization of behaviour in pairs: the role of communication and consequences in timing. *Anim. Behav.* 74 1735–1742. 10.1016/j.anbehav.2007.04.014

[B50] Duranton C. Gaunet F. (2016). Behavioural synchronization from an ethological perspective: overview of its adaptive value. *Adapt. Behav.* 24 181–191. 10.1177/1059712316644966

[B51] Durkheim E. (1912). *The Elementary Forms of Religious Life.* Paris: Alcan.

[B52] Elie J. E. Mariette M. M. Soula H. A. Griffith S. C. Mathevon N. Vignal C. (2010). Vocal communication at the nest between mates in wild zebra finches: a private vocal duet? *Anim. Behav.* 80 597–605. 10.1016/j.anbehav.2010.06.003

[B53] Elie J. E. Mathevon N. Vignal C. (2011a). Same-sex pair-bonds are equivalent to male–female bonds in a life-long socially monogamous songbird. *Behav. Ecol. Sociobiol.* 65 2197–2208. 10.1007/s00265-011-1228-9

[B54] Elie J. E. Soula H. A. Mathevon N. Vignal C. (2011b). Dynamics of communal vocalizations in a social songbird, the zebra finch (*Taeniopygia guttata*). *J. Acoust. Soc. Am.* 129 4037–4046. 10.1121/1.3570959 21682424

[B55] Emery Thompson M. (2019). How can non-human primates inform evolutionary perspectives on female-biased kinship in humans? *Philos. Trans. R. Soc. B* 374 20180074. 10.1098/rstb.2018.0074 31303156 PMC6664131

[B56] Ens B. J. Choudhury S. Black J. M. (1996). “Mate fidelity and divorce in monogamous birds,” in *Partnerships in Birds: The Study of Monogamy: The Study of Monogamy*, ed. Black J. M. (Oxford: Oxford University Press), 344–401.

[B57] Feldman R. (2007). Parent–infant synchrony and the construction of shared timing; physiological precursors, developmental outcomes, and risk conditions. *J. Child Psychol. Psychiatry* 48 329–354. 10.1111/j.1469-7610.2006.01701.x 17355401

[B58] Feldman R. (2012a). Bio-behavioral synchrony: a model for integrating biological and microsocial behavioral processes in the study of parenting. *Parenting* 12 154–164. 10.1080/15295192.2012.683342

[B59] Feldman R. (2012b). Oxytocin and social affiliation in humans. *Horm. Behav.* 61 380–391. 10.1016/j.yhbeh.2012.01.008 22285934

[B60] Feldman R. (2015). The adaptive human parental brain: implications for children’s social development. *Trends Neurosci.* 38 387–399. 10.1016/j.tins.2015.04.004 25956962

[B61] Feldman R. Gordon I. Zagoory-Sharon O. (2011). Maternal and paternal plasma, salivary, and urinary oxytocin and parent–infant synchrony: considering stress and affiliation components of human bonding. *Dev. Sci.* 14 752–761. 10.1111/j.1467-7687.2010.01021.x 21676095

[B62] Feldman R. Weller A. Zagoory-Sharon O. Levine A. (2007). Evidence for a neuroendocrinological foundation of human affiliation: plasma oxytocin levels across pregnancy and the postpartum period predict mother-infant bonding. *Psychol. Sci.* 18 965–970. 10.1111/j.1467-9280.2007.02010.x 17958710

[B63] Fernandez M. S. A. Vignal C. Soula H. A. (2017). Impact of group size and social composition on group vocal activity and acoustic network in a social songbird. *Anim. Behav.* 127 163–178. 10.1016/j.anbehav.2017.03.013

[B64] Finkenwirth C. Burkart J. M. (2017). Long-term-stability of relationship structure in family groups of common marmosets, and its link to proactive prosociality. *Physiol. Behav.* 173 79–86. 10.1016/j.physbeh.2017.01.032 28115225

[B65] Finkenwirth C. Burkart J. M. (2018). Why help? Relationship quality, not strategic grooming predicts infant-care in group-living marmosets. *Physiol. Behav.* 193 108–116. 10.1016/j.physbeh.2018.02.050 29730031

[B66] Finkenwirth C. van Schaik C. Ziegler T. E. Burkart J. M. (2015). Strongly bonded family members in common marmosets show synchronized fluctuations in oxytocin. *Physiol. Behav.* 151 246–251. 10.1016/j.physbeh.2015.07.034 26232089 PMC5916785

[B67] Fleming A. S. O’Day D. H. Kraemer G. W. (1999). Neurobiology of mother–infant interactions: experience and central nervous system plasticity across development and generations. *Neurosci. Biobehav. Rev.* 23 673–685. 10.1016/s0149-7634(99)00011-1 10392659

[B68] Focardi S. Pecchioli E. (2005). Social cohesion and foraging decrease with group size in fallow deer (Dama dama). *Behav. Ecol. Sociobiol.* 59 84–91. 10.1007/s00265-005-0012-0

[B69] Fortune E. S. Rodríguez C. Li D. Ball G. F. Coleman M. J. (2011). Neural mechanisms for the coordination of duet singing in wrens. *Science* 334 666–670. 10.1126/science.1209867 22053048

[B70] Frigerio D. Weiss B. Kotrschal K. (2001). Spatial proximity among adult siblings in greylag geese (*Anser anser*): evidence for female bonding? *Acta Ethol.* 3 121–125. 10.1007/s102110000028

[B71] Gill L. F. Goymann W. Ter Maat A. Gahr M. L. (2015). Patterns of call communication between group-housed zebra finches change during the breeding cycle. *eLife* 4:e07770. 10.7554/eLife.07770 PMC4592938 26441403

[B72] Gordon I. Zagoory-Sharon O. Leckman J. F. Feldman R. (2010). Oxytocin and the development of parenting in humans. *Biol. Psychiatry* 68 377–382. 10.1016/j.biopsych.2010.02.005 20359699 PMC3943240

[B73] Gorissen L. Eens M. (2004). Interactive communication between male and female great tits (*Parus major*) during the dawn chorus. *Auk* 121 184–191. 10.1093/auk/121.1.184

[B74] Gowaty P. A. (1996). “Battles of the sexes and origins of monogamy,” in *Partnerships in Birds*, ed. Black J. M. (Oxford: Oxford University Press), 21–52.

[B75] Grammer K. Honda M. Juette A. Schmitt A. (1999). Fuzziness of nonverbal courtship communication unblurred by motion energy detection. *J. Personal. Soc. Psychol.* 77 487–508. 10.1037/0022-3514.77.3.487 10510505

[B76] Greenberg R. S. (2001). “Birds of many feathers: the formation and structure of mixed species flocks of forest birds,” in *On the Move: How and Why Animals Travel in Groups*, eds Boinski S. Garber P. A. (Chicago, IL: University of Chicago Press), 521–558.

[B77] Griggio M. Hoi H. (2011). An experiment on the function of the long-term pair bond period in the socially monogamous bearded reedling. *Anim. Behav.* 82 1329–1335. 10.1016/j.anbehav.2011.09.016

[B78] Gueguen N. Jacob C. Martin A. (2009). Mimicry in social interaction: its effect on human judgment and behavior. *Eur. J. Soc. Sci.* 8 253–259.

[B79] Hall M. L. (2009). A review of vocal duetting in birds. *Adv. Study Behav.* 40 67–121. 10.1016/s0065-3454(09)40003-2

[B80] Hall M. L. Magrath R. D. (2007). Temporal coordination signals coalition quality. *Curr. Biol.* 17 R406–R407. 17550763 10.1016/j.cub.2007.04.022

[B81] Handegard N. O. Boswell K. M. Ioannou C. C. Leblanc S. P. Tjøstheim D. B. Couzin I. D. (2012). The dynamics of coordinated group hunting and collective information transfer among schooling prey. *Curr. Biol.* 22 1213–1217. 10.1016/j.cub.2012.04.050 22683262

[B82] Hasson U. (2016). Face to face, brain to brain: exploring the mechanisms of dyadic social interactions. *Int. J. Psychol.* 51 873–874.

[B83] Hasson U. Ghazanfar A. A. Galantucci B. Garrod S. Keysers C. (2012). Brain-to-brain coupling: a mechanism for creating and sharing a social world. *Trends Cogn. Sci.* 16 114–121. 10.1016/j.tics.2011.12.007 22221820 PMC3269540

[B84] Hatfield E. Cacioppo J. T. Rapson R. L. (1993). Emotional contagion. *Cur. Dir. Psychol. Sci.* 2 96–100.

[B85] Helm J. L. Sbarra D. Ferrer E. (2012). Assessing cross-partner associations in physiological responses via coupled oscillator models. *Emotion* 12 748–762. 10.1037/a0025036 21910541

[B86] Hinde C. A. Kilner R. M. (2006). Negotiations within the family over the supply of parental care. *Proc. R. Soc. B Biol. Sci.* 274 53–60. 10.1098/rspb.2006.3692 17015339 PMC1679882

[B87] Hinde R. A. (1952). The behaviour of the great tit (*Parus major*) and some other related species. *Behav. Suppl.* 2 1–20.

[B88] Hirschenhauser K. (2012). Testosterone and partner compatibility: evidence and emerging questions. *Ethology* 118 799–811. 10.1111/j.1439-0310.2012.02087.x

[B89] Hirschenhauser K. Mostl E. Kotrschal K. (1999). Within-pair testosterone covariation and reproductive output in Greylag Geese *Anser anser*. *Ibis* 141 577–586. 10.1111/j.1474-919x.1999.tb07365.x

[B90] Hirschenhauser K. Weiß B. M. Haberl W. Möstl E. Kotrschal K. (2010). Female androgen patterns and within-pair testosterone compatibility in domestic geese (*Anser domesticus*). *Gen. Comp. Endocrinol.* 165 195–203. 10.1016/j.ygcen.2009.06.022 19576216

[B91] Hughes C. (1998). Integrating molecular techniques with field methods in studies of social behavior: a revolution results. *Ecology* 79 383–399. 10.1890/0012-9658(1998)079[0383:imtwfm]2.0.co;2

[B92] Ihle M. Kempenaers B. Forstmeier W. (2015). Fitness benefits of mate choice for compatibility in a socially monogamous species. *PLoS Biol.* 13:e1002248. 10.1371/journal.pbio.1002248 26366558 PMC4569426

[B93] Jaffé R. Pioker-Hara F. C. dos Santos C. F. Santiago L. R. Alves D. A. de Mp A. (2014). Monogamy in large bee societies: a stingless paradox. *Naturwissenschaften* 101 261–264. 10.1007/s00114-014-1149-3 24463620

[B94] Johnstone R. A. Manica A. Fayet A. L. Stoddard M. C. Rodriguez-Gironés M. A. Hinde C. A. (2013). Reciprocity and conditional cooperation between great tit parents. *Behav. Ecol.* 25 216–222. 10.1093/beheco/art109 27193460

[B95] Kelso J. A. S. (1995). *Dynamic Patterns: The Self-Organization of Brain and Behavior.* Cambridge, MA: MIT press.

[B96] Kendon A. (1970). Movement coordination in social interaction: Some examples described. *Acta Psychol.* 32 101–125. 10.1016/0001-6918(70)90094-6 5444439

[B97] Kenny E. Birkhead T. R. Green J. P. (2017). Allopreening in birds is associated with parental cooperation and stable pair bonds across years. *Behav. Ecol.* 28 1142–1148. 10.1093/beheco/arx078 29622926 PMC5873249

[B98] King A. J. Cowlishaw G. (2009). All together now: behavioural synchrony in baboons. *Anim. Behav.* 78 1381–1387. 10.1016/j.anbehav.2009.09.009

[B99] Kinreich S. Djalovski A. Kraus L. Louzoun Y. Feldman R. (2017). Brain-to-brain synchrony during naturalistic social interactions. *Sci. Rep.* 7:17060. 10.1038/s41598-017-17339-5 29213107 PMC5719019

[B100] Kleiman D. G. (1977). Monogamy in mammals. *Q. Rev. Biol.* 52 39–69. 10.1086/409721 857268

[B101] Koehne S. Hatri A. Cacioppo J. T. Dziobek I. (2016). Perceived interpersonal synchrony increases empathy: insights from autism spectrum disorder. *Cognition* 146 8–15. 10.1016/j.cognition.2015.09.007 26398860

[B102] Koudenburg N. Postmes T. Gordijn E. H. (2017). Beyond content of conversation: the role of conversational form in the emergence and regulation of social structure. *Personal. Soc. Psychol. Rev.* 21 50–71. 10.1177/1088868315626022 26874307

[B103] Lack D. L. (1968). *Ecological Adaptations for Breeding in Birds.* London: Methuen.

[B104] Lakin J. L. Chartrand T. L. (2003). Using nonconscious behavioral mimicry to create affiliation and rapport. *Psychol. Sci.* 14 334–339. 10.1111/1467-9280.14481 12807406

[B105] Laubu C. Dechaume-Moncharmont F.-X. Motreuil S. Schweitzer C. (2016). Mismatched partners that achieve postpairing behavioral similarity improve their reproductive success. *Sci. Adv.* 2:e1501013. 10.1126/sciadv.1501013 26973869 PMC4783125

[B106] Leniowski K. Węgrzyn E. (2018). Synchronisation of parental behaviours reduces the risk of nest predation in a socially monogamous passerine bird. *Sci. Rep.* 8:7385. 10.1038/s41598-018-25746-5 29743657 PMC5943351

[B107] Levenson R. W. Gottman J. M. (1983). Marital interaction: physiological linkage and affective exchange. *J. Personal. Soc. Psychol.* 45 587–597. 10.1037/0022-3514.45.3.587 6620126

[B108] Liker A. Székely T. (1999). Mating pattern and mate choice in the lapwing vanellus vanellus. *Ornis Hungarica* 8 13–25.

[B109] Liu Y. Piazza E. A. Simony E. Shewokis P. A. Onaral B. Hasson U. (2017). Measuring speaker–listener neural coupling with functional near infrared spectroscopy. *Sci. Rep.* 7:43293. 10.1038/srep43293 28240295 PMC5327440

[B110] Llobera J. Charbonnier C. Chagué S. Preissmann D. Antonietti J.-P. Ansermet F. (2016). The subjective sensation of synchrony: an experimental study. *PLoS One* 11:e0147008. 10.1371/journal.pone.0147008 26870943 PMC4752214

[B111] Lukas D. Clutton-Brock T. H. (2013). The evolution of social monogamy in mammals. *Science* 341 526–530. 10.1126/science.1238677 23896459

[B112] Lumsden J. Miles L. K. Richardson M. J. Smith C. A. Macrae C. N. (2012). Who syncs? Social motives and interpersonal coordination. *J. Exp. Soc. Psychol.* 48 746–751. 10.1016/j.jesp.2011.12.007

[B113] Mainwaring M. C. Griffith S. C. (2013). Looking after your partner: sentinel behaviour in a socially monogamous bird. *PeerJ* 1:e83. 10.7717/peerj.83 23761856 PMC3678116

[B114] Manica L. T. Graves J. A. Podos J. Macedo R. H. (2016). Multimodal flight display of a neotropical songbird predicts social pairing but not extrapair mating success. *Behav. Ecol. Sociobiol.* 70 2039–2052. 10.1007/s00265-016-2208-x

[B115] Mariette M. M. (2019). Acoustic cooperation: acoustic communication regulates conflict and cooperation within the family. *Front. Ecol. Evol.* 7:445 10.3389/fevo.2019.00445

[B116] Mariette M. M. Griffith S. C. (2012). Nest visit synchrony is high and correlates with reproductive success in the wild zebra finch *Taeniopygia guttata*. *J. Avian Biol.* 43 131–140. 10.1111/j.1600-048x.2012.05555.x

[B117] Mariette M. M. Griffith S. C. (2015). The adaptive significance of provisioning and foraging coordination between breeding partners. *Am. Nat.* 185 270–280. 10.1086/679441 25616144

[B118] Mazzurega M. Pavani F. Paladino M. P. Schubert T. W. (2011). Self-other bodily merging in the context of synchronous but arbitrary-related multisensory inputs. *Exp. Brain Res.* 213 213–221. 10.1007/s00221-011-2744-6 21656218

[B119] McDowall J. J. (1978). Interactional synchrony: a reappraisal. *J. Personal. Soc. Psychol.* 36 963–975. 10.1037/0022-3514.36.9.963

[B120] Miles L. K. Griffiths J. L. Richardson M. J. Macrae C. N. (2010). Too late to coordinate: contextual influences on behavioral synchrony. *Eur. J. Soc. Psychol.* 40 52–60. 10.1002/ejsp.721

[B121] Moore S. D. Rohwer V. G. (2012). The functions of adult female begging during incubation in sub-Arctic breeding yellow warblers. *Anim. Behav.* 84 1213–1219. 10.1016/j.anbehav.2012.08.027

[B122] Morley J. I. Balshine S. (2002). Faithful fish: territory and mate defence favour monogamy in an African cichlid fish. *Behav. Ecol. Sociobiol.* 52 326–331. 10.1007/s00265-002-0520-0

[B123] Mu Y. Han S. Gelfand M. J. (2017). The role of gamma interbrain synchrony in social coordination when humans face territorial threats. *Soc. Cogn. Affect. Neurosci.* 12 1614–1623. 10.1093/scan/nsx093 28985437 PMC5647809

[B124] Mulder M. B. (2009). Serial monogamy as polygyny or polyandry? *Hum. Nat.* 20 130–150. 10.1007/s12110-009-9060-x 25526955 PMC5486523

[B125] Nalepa C. A. Jones S. C. (1991). Evolution of monogamy in termites. *Biol. Rev.* 66 83–97. 10.1111/j.1469-185x.1991.tb01136.x

[B126] Niebuhr V. McFarland D. (1983). Nest-relief behaviour in the herring gull. *Anim. Behav.* 31 701–707. 10.1016/s0003-3472(83)80225-5

[B127] O’Connell L. A. Hofmann H. A. (2012). Evolution of a vertebrate social decision-making network. *Science* 336 1154–1157. 10.1126/science.1218889 22654056

[B128] Odom K. J. Omland K. E. (2017). Females and males respond more strongly to duets than to female solos: comparing the function of duet and solo singing in a tropical songbird (*Icterus icterus*). *Behaviour* 154 1377–1395. 10.1163/1568539x-00003473

[B129] Ophir A. G. Phelps S. M. Sorin A. B. Wolff J. O. (2008). Social but not genetic monogamy is associated with greater breeding success in prairie voles. *Anim. Behav.* 75 1143–1154. 10.1016/j.anbehav.2007.09.022

[B130] Ota N. Gahr M. Soma M. (2015). Tap dancing birds: the multimodal mutual courtship display of males and females in a socially monogamous songbird. *Sci. Rep.* 5:16614. 10.1038/srep16614 26583485 PMC4994120

[B131] Ota N. Gahr M. Soma M. (2018). Couples showing off: audience promotes both male and female multimodal courtship display in a songbird. *Sci. Adv.* 4:eaat4779. 10.1126/sciadv.aat4779 30306131 PMC6170041

[B132] Otter K. A. Atherton S. E. Van Oort H. (2007). Female food solicitation calling, hunger levels and habitat differences in the black-capped chickadee. *Anim. Behav.* 74 847–853. 10.1016/j.anbehav.2007.01.016

[B133] Ouyang J. Q. Van Oers K. Quetting M. Hau M. (2014). Becoming more like your mate: hormonal similarity reduces divorce rates in a wild songbird. *Anim. Behav.* 98 87–93. 10.1016/j.anbehav.2014.09.032

[B134] Paladino M.-P. Mazzurega M. Pavani F. Schubert T. W. (2010). Synchronous multisensory stimulation blurs self-other boundaries. *Psychol. Sci.* 21 1202–1207. 10.1177/0956797610379234 20679523

[B135] Paxton A. Dale R. (2013). *Argument Disrupts Interpersonal Synchrony.* London: SAGE Publications. 10.1080/17470218.2013.853089 24303888

[B136] Pays O. Jarman P. J. Loisel P. Gerard J.-F. (2007). Coordination, independence or synchronization of individual vigilance in the eastern grey kangaroo? *Anim. Behav.* 73 595–604. 10.1016/j.anbehav.2006.06.007

[B137] Perfito N. Zann R. A. Bentley G. E. Hau M. (2007). Opportunism at work: habitat predictability affects reproductive readiness in free-living zebra finches. *Funct. Ecol.* 21 291–301. 10.1111/j.1365-2435.2006.01237.x

[B138] Preissmann D. Charbonnier C. Chagué S. Antonietti J.-P. Llobera J. Ansermet F. (2016). A motion capture study to measure the feeling of synchrony in romantic couples and in professional musicians. *Front. Psychol.* 7:1673. 10.3389/fpsyg.2016.01673 27833580 PMC5082227

[B139] Prior N. H. Fernandez M. S. A. Soula H. A. Vignal C. (2018). Rapid effects of sex steroids on zebra finch (*Taeniopygia guttata*) pair maintenance. *Behav. Neurosci.* 132 536–546. 10.1037/bne0000263 30284861

[B140] Prior N. H. Heimovics S. A. Soma K. K. (2013). Effects of water restriction on reproductive physiology and affiliative behavior in an opportunistically-breeding and monogamous songbird, the zebra finch. *Horm. Behav.* 63 462–474. 10.1016/j.yhbeh.2012.12.010 23274698

[B141] Prior N. H. Smith E. Dooling R. J. Ball G. F. (2019). Familiarity enhances moment-to-moment behavioral coordination in zebra finch (*Taeniopygia guttata*) dyads. *J. Comp. Psychol.* 134 135–148. 10.1037/com0000201 31647250 PMC7180088

[B142] Prior N. H. Soma K. K. (2015). Neuroendocrine regulation of long-term pair maintenance in the monogamous zebra finch. *Horm. Behav.* 76 11–22. 10.1016/j.yhbeh.2015.04.014 25935729

[B143] Prior N. H. Smith E. Dooling R. J. Ball G. F. (2020). *Monogamy in a moment: how do brief social interactions change over time in pair-bonded Zebra Finches (Taeniopygia guttata)? bioRxiv* [Preprint]. 10.1101/2020.06.18.160051 PMC7810576 33791572

[B144] Prior N. H. Yap K. N. Liu T. Q. D. Vignal C. Soma K. K. (2016). Context-dependent effects of testosterone treatment to males on pair maintenance behaviour in zebra finches. *Anim. Behav.* 114 155–164. 10.1016/j.anbehav.2016.01.023

[B145] Prior N. H. Yap K. N. Soma K. K. (2014). Acute and chronic effects of an aromatase inhibitor on pair-maintenance behavior of water-restricted zebra finch pairs. *Gen. Comp. Endocrinol.* 196 62–71. 10.1016/j.ygcen.2013.10.018 24231681

[B146] Raihani N. J. Nelson-Flower M. J. Moyes K. Browning L. E. Ridley A. R. (2010). Synchronous provisioning increases brood survival in cooperatively breeding pied babblers. *J. Anim. Ecol.* 79 44–52. 10.1111/j.1365-2656.2009.01606.x 19674178

[B147] Ramseyer F. Tschacher W. (2011). Nonverbal synchrony in psychotherapy: coordinated body movement reflects relationship quality and outcome. *J. Consult. Clin. Psychol.* 79 284–295. 10.1037/a0023419 21639608

[B148] Rangassamy M. Dalmas M. Féron C. Gouat P. Rödel H. G. (2015). Similarity of personalities speeds up reproduction in pairs of a monogamous rodent. *Anim. Behav.* 103 7–15. 10.1016/j.anbehav.2015.02.007

[B149] Reddish P. Tong E. M. W. Jong J. Whitehouse H. (2020). Interpersonal synchrony affects performers’ sense of agency. *Self Identity* 19 389–411. 10.1080/15298868.2019.1604427

[B150] Reichard U. H. (2003). *Monogamy: Past and Present. Monogamy: Mating Strategies and Partnerships in Birds Humans and Other Mammals.* Cambridge: Cambridge University Press, 3–25.

[B151] Reichard U. H. Boesch C. (2003). *Monogamy: Mating Strategies and Partnerships in Birds, Humans and Other Mammals.* Cambridge: Cambridge University Press.

[B152] Ręk P. Wong B. (2017). Multimodal coordination enhances the responses to an avian duet. *Behav. Ecol.* 29 411–417. 10.1093/beheco/arx174 27193460

[B153] Rennung M. Göritz A. S. (2016). Prosocial consequences of interpersonal synchrony. *Z. Psychol.* 224 168–189. 10.1027/2151-2604/a000252 28105388 PMC5137339

[B154] Resendez S. L. Keyes P. C. Day J. J. Hambro C. Austin C. J. Maina F. K. (2016). Dopamine and opioid systems interact within the nucleus accumbens to maintain monogamous pair bonds. *eLife* 5:e15325 10.7554/eLife.15325.001 PMC4972541 27371827

[B155] Sakai M. Morisaka T. Kogi K. Hishii T. Kohshima S. (2010). Fine-scale analysis of synchronous breathing in wild indo-pacific bottlenose dolphins (*Tursiops aduncus*). *Behav. Process.* 83 48–53. 10.1016/j.beproc.2009.10.001 19850113

[B156] Savage J. L. Browning L. E. Manica A. Russell A. F. Johnstone R. A. (2017). Turn-taking in cooperative offspring care: by-product of individual provisioning behavior or active response rule? *Behav. Ecol. Sociobiol.* 71:162. 10.1007/s00265-017-2391-4 29081573 PMC5644705

[B157] Scheele D. Wille A. Kendrick K. M. Stoffel-Wagner B. Becker B. Güntürkün O. (2013). Oxytocin enhances brain reward system responses in men viewing the face of their female partner. *Proc. Natl. Acad. Sci. U.S.A.* 110 20308–20313. 10.1073/pnas.1314190110 24277856 PMC3864312

[B158] Scheflen A. E. (1982). “Comments on the significance of interaction rhythms. Interaction rhythms,” in *Periodicity in Communicative Behavior*, ed. Davis M. (New York, NY: Human Sciences Press), 13–22.

[B159] Schneiderman I. Zagoory-Sharon O. Leckman J. F. Feldman R. (2012). Oxytocin during the initial stages of romantic attachment: relations to couples’ interactive reciprocity. *Psychoneuroendocrinology* 37 1277–1285. 10.1016/j.psyneuen.2011.12.021 22281209 PMC3936960

[B160] Scribner J. L. Vance E. Protter D. S. W. Saslow E. Cameron R. Klein E. (2019). A neuronal signature for monogamous reunion. *BioRxiv* [Preprint]. 10.1101/675959 PMC7245077 32381740

[B161] Sharon-David H. Mizrahi M. Rinott M. Golland Y. Birnbaum G. E. (2018). Being on the same wavelength: behavioral synchrony between partners and its influence on the experience of intimacy. *J. Soc. Pers. Relationsh.* 36 2983–3008. 10.1177/0265407518809478

[B162] Shen S.-F. Chen H.-C. Vehrencamp S. L. Yuan H.-W. (2010). Group provisioning limits sharing conflict among nestlings in joint-nesting Taiwan yuhinas. *Biol. Lett.* 6 318–321. 10.1098/rsbl.2009.0909 20053663 PMC2880055

[B163] Silver R. (1983). *Biparental Care in Birds: Mechanisms Controlling Incubation Bout Duration, Hormones and Behaviour in Higher Vertebrates.* Berlin: Springer, 451–462.

[B164] Silver R. Andrews H. Ball G. F. (1985). Parental care in an ecological perspective: a quantitative analysis of avian subfamilies. *Am. Zool.* 25 823–840. 10.1093/icb/25.3.823 31919651

[B165] Sládeček M. Vozabulová E. Brynychová K. Šálek M. E. (2019). Parental incubation exchange in a territorial bird species involves sex-specific signalling. *Front. Zool.* 16:7. 10.1186/s12983-019-0306-0 30949226 PMC6431054

[B166] Solomon N. G. Keane B. Knoch L. R. Hogan P. J. (2004). Multiple paternity in socially monogamous prairie voles (*Microtus ochrogaster*). *Can. J. Zool.* 82 1667–1671. 10.1139/z04-142

[B167] Soma M. Garamszegi L. Z. (2015). Evolution of courtship display in Estrildid finches: dance in relation to female song and plumage ornamentation. *Front. Ecol. Evol.* 3:4 10.3389/fevo.2015.00004

[B168] Spoon T. R. Millam J. R. Owings D. H. (2006). The importance of mate behavioural compatibility in parenting and reproductive success by cockatiels. *Nymphicus hollandicus*. *Anim. Behav.* 71 315–326. 10.1016/j.anbehav.2005.03.034

[B169] Szipl G. Loth A. Wascher C. A. F. Hemetsberger J. Kotrschal K. Frigerio D. (2019). Parental behaviour and family proximity as key to gosling survival in Greylag Geese (*Anser anser*). *J. Ornithol.* 160 473–483. 10.1007/s10336-019-01638-x 31098339 PMC6476843

[B170] Takeda K. F. Hiraiwa-Hasegawa M. Kutsukake N. (2018). Uncoordinated dances associated with high reproductive success in a crane. *Behav. Ecol.* 30 101–106. 10.1093/beheco/ary159 27193460

[B171] Tarr B. Launay J. Dunbar R. I. M. (2016). Silent disco: dancing in synchrony leads to elevated pain thresholds and social closeness. *Evol. Hum. Behav.* 37 343–349. 10.1016/j.evolhumbehav.2016.02.004 27540276 PMC4985033

[B172] Tecot S. R. Singletary B. Eadie E. (2016). Why “monogamy” isn’t good enough. *Am. J. Primatol.* 78 340–354. 10.1002/ajp.22412 25864507

[B173] Tobias J. A. Seddon N. (2002). Female begging in European robins: do neighbors eavesdrop for extrapair copulations? *Behav. Ecol.* 13 637–642. 10.1093/beheco/13.5.637

[B174] Tomaszycki M. L. Adkins-Regan E. (2005). Experimental alteration of male song quality and output affects female mate choice and pair bond formation in zebra finches. *Anim. Behav.* 70 785–794. 10.1016/j.anbehav.2005.01.010

[B175] Tomaszycki M. L. Adkins-Regan E. (2006). Is male song quality important in maintaining pair bonds? *Behaviour* 143 549–567. 10.1163/156853906776759529

[B176] Tomaszycki M. L. Zatirka B. P. (2014). Same-sex partner preference in zebra finches: pairing flexibility and choice. *Arch. Sex. Behav.* 43 1469–1475. 10.1007/s10508-014-0377-0 25190500

[B177] Tunçgenç B. Cohen E. (2016). Movement synchrony forges social bonds across group divides. *Front. Psychol.* 7:782. 10.3389/fpsyg.2016.00782 27303341 PMC4882973

[B178] Ulmer-Yaniv A. Avitsur R. Kanat-Maymon Y. Schneiderman I. Zagoory-Sharon O. Feldman R. (2016). Affiliation, reward, and immune biomarkers coalesce to support social synchrony during periods of bond formation in humans. *Brain Behav. Immun.* 56 130–139. 10.1016/j.bbi.2016.02.017 26902915

[B179] Valdesolo P. DeSteno D. (2011). Synchrony and the social tuning of compassion. *Emotion* 11 262–266. 10.1037/a0021302 21500895

[B180] Van Baaren R. B. Holland R. W. Kawakami K. Van Knippenberg A. (2004). Mimicry and prosocial behavior. *Psychol. Sci.* 15 71–74. 10.1111/j.0963-7214.2004.01501012.x 14717835

[B181] van Rooij E. P. Griffith S. C. (2013). Synchronised provisioning at the nest: parental coordination over care in a socially monogamous species. *PeerJ* 1:e232. 10.7717/peerj.232 24432197 PMC3883492

[B182] Vicaria I. M. Dickens L. (2016). Meta-analyses of the intra-and interpersonal outcomes of interpersonal coordination. *J. Nonverb. Behav.* 40 335–361. 10.1007/s10919-016-0238-8

[B183] Villain A. S. Fernandez M. S. A. Bouchut C. Soula H. A. Vignal C. (2016). Songbird mates change their call structure and intrapair communication at the nest in response to environmental noise. *Anim. Behav.* 116 113–129. 10.1016/j.anbehav.2016.03.009

[B184] Villain A. S. Mahamoud-Issa M. Doligez B. Vignal C. (2017). Vocal behaviour of mates at the nest in the white-throated dipper cinclus cinclus: contexts and structure of vocal interactions, pair-specific acoustic signature. *J. Ornithol.* 158 897–910. 10.1007/s10336-017-1449-4

[B185] Wachtmeister C.-A. (2001). Display in monogamous pairs: a review of empirical data and evolutionary explanations. *Anim. Behav.* 61 861–868. 10.1006/anbe.2001.1684

[B186] Wallman J. Grabon M. Silver R. (1979). What determines the pattern of sharing of incubation and brooding in ring doves? *J. Comp. Physiol. Psychol.* 93 481–492. 10.1037/h0077576

[B187] Whiteman E. A. Côté I. M. (2004). Monogamy in marine fishes. *Biol. Rev.* 79 351–375. 10.1017/s1464793103006304 15191228

[B188] Wickler W. Seibt U. (1981). Monogamy in crustacea and man. *Z. Tierpsychol.* 57 215–234. 10.1111/j.1439-0310.1981.tb01924.x

[B189] Williams J. R. Catania K. C. Carter C. S. (1992). Development of partner preferences in female prairie voles (*Microtus ochrogaster*): the role of social and sexual experience. *Horm. Behav.* 26 339–349. 10.1016/0018-506x(92)90004-f 1398553

[B190] Wiltermuth S. S. Heath C. (2009). Synchrony and cooperation. *Psychol. Sci.* 20 1–5. 10.1111/j.1467-9280.2008.02253.x 19152536

[B191] Wojczulanis-Jakubas K. Araya-Salas M. Jakubas D. (2018). Seabird parents provision their chick in a coordinated manner. *PLoS One* 13:e0189969. 10.1371/journal.pone.0189969 29320525 PMC5761830

[B192] Wundt W. (1894). Lectures on human and animal psychology”. Translated by JE Creighton and EB Titchener. *Ancient Philos.* 5:631.

[B193] Yorio P. Boersma P. D. (1994). Causes of nest desertion during incubation in the magellanic penguin (*Spheniscus magellanicus*). *Condor* 96 1076–1083. 10.2307/1369116

[B194] Young L. J. Wang Z. (2004). The neurobiology of pair bonding. *Nat. Neurosci.* 7 1048–1054. 10.1038/nn1327 15452576

[B195] Zann R. A. (1996). *The Zebra Finch: a Synthesis of Field and Laboratory Studies.* Oxford: Oxford University Press.

